# Supplementary material for: Optimizing AAV2/6 microglial targeting identified enhanced efficiency in the photoreceptor degenerative environment
Source: Mol Ther Methods Clin Dev. 2021 Sep 14;23:210–24. doi: 10.1016/j.omtm.2021.09.006 (PMC8516996; doi:10.1016/j.omtm.2021.09.006)
Supplement: Document S1. Figures S1–S8 [file mmc1.pdf]

OMTM, Volume *23*

Supplemental information

Optimizing AAV2/6 microglial targeting identified enhanced efficiency in the photoreceptor degenerative environment

Margaret E. Maes, Gabriele M. Wögenstein, Gloria Colombo, Raquel Casado-Polanco, and Sandra Siegert

Figure S1

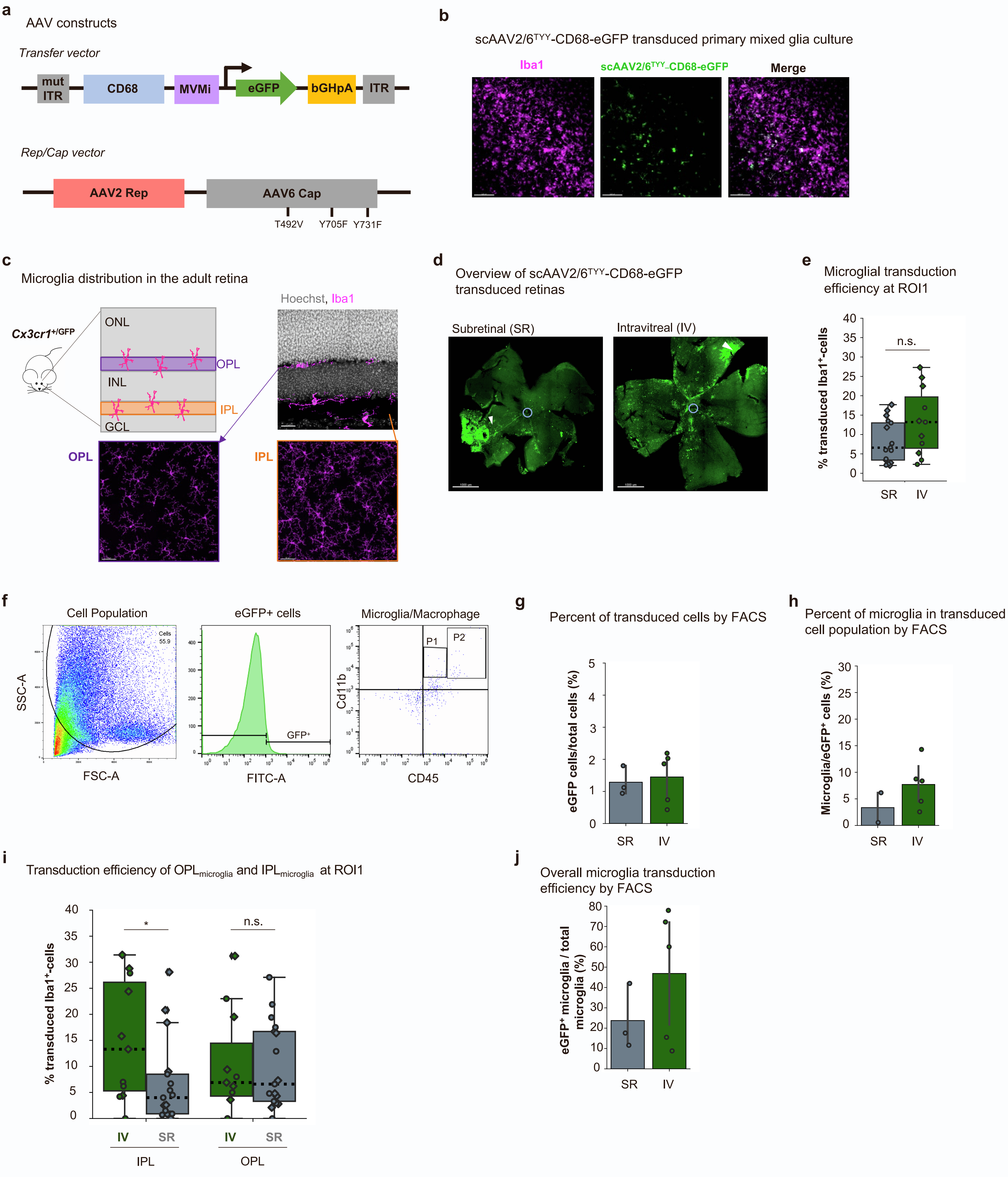

Figure S1. scAAV2/6^{TYY}-CD68-eGFP design and validation *in vitro* and *in vivo*

(**a**) Transfer vector illustration containing the *CD68* promoter, MVM intron and Rep/Cap vector with indicated TYY mutations in capsid sequence. (**b**) Mixed primary glia cultures transduced with 1*10^{8} viral genomes/well of scAAV2/6^{TYY}-CD68-eGFP (green) counterstained with Iba1 (magenta). Scale bar: 200µm. (**c**) Pictorial representation of the retinal layers, highlighting the outer plexiform layer (OPL, purple) and inner plexiform layer (IPL, orange), with corresponding confocal images of retinal cross section or whole mount images of Iba1-stained microglia (magenta). Scale bar: 50µm. (**d**) Overview images of retinas from subretinal (SR) or intravitreal (IV) injection indicating viral-mediated eGFP transgene expression. Blue circle indicates optic nerve head. White triangles: injection site. (**e**) Microglial transduction efficiency from quantification from ROI1 in both plexiform layers using 20X images. (Wilcoxon rank-sum test: *P* = 0.011). Scale bar: 1000µm. (**f**) Gating strategy for FACS sorted eGFP^{+} cells from a transduced retina. Microglia (P1) are CD11b^{hi}/CD45^{lo}, while macrophages (P2) are CD11b^{hi}/CD45^{hi}. (**g**) Percentage of eGFP^{+} cells in total cell population or (**h**) percent of transduced eGFP^{+} cells that are microglia in intravitreal and subretinal injected retinas. (IV: n= 5 retinas, 3 mice; SR: n = 3 retinas, 3 mice) (**i**) Inter-layer comparison of microglia transduction efficiency at ROI1. (Wilcoxon rank-sum test: IPL SR-IV, *P* = 0.048; OPL SR-IV, *P* = 0.79). (**j**) Overall microglia transduction efficiency. Percent of eGFP^{+} CD11b^{hi}/CD45^{lo} microglia / total CD11b^{hi}/CD45^{lo} microglia. *P < 0.05, ^{ns}P > 0.05. ITR, inverted terminal repeat; *CD68*, Cluster of differentiation 68; MVMi, minute virus of mice intron; eGFP, enhanced green fluorescent protein; bGHpA, bovine growth hormone polyadenylation signal; Rep, Replication; Cap, Capsid; scAAV, self-complementary adeno-associated virus; Iba1, ionized calcium binding adaptor molecule 1; ONL, outer nuclear layer; OPL, outer plexiform layer INL, inner nuclear layer; IPL, inner plexiform layer; GCL, ganglion cell layer; Cx3cr1, CX3C chemokine receptor 1; SR, subretinal; IV, intravitreal; P, postnatal day; ROI, region of interest; n.s., not significant.

Figure S2

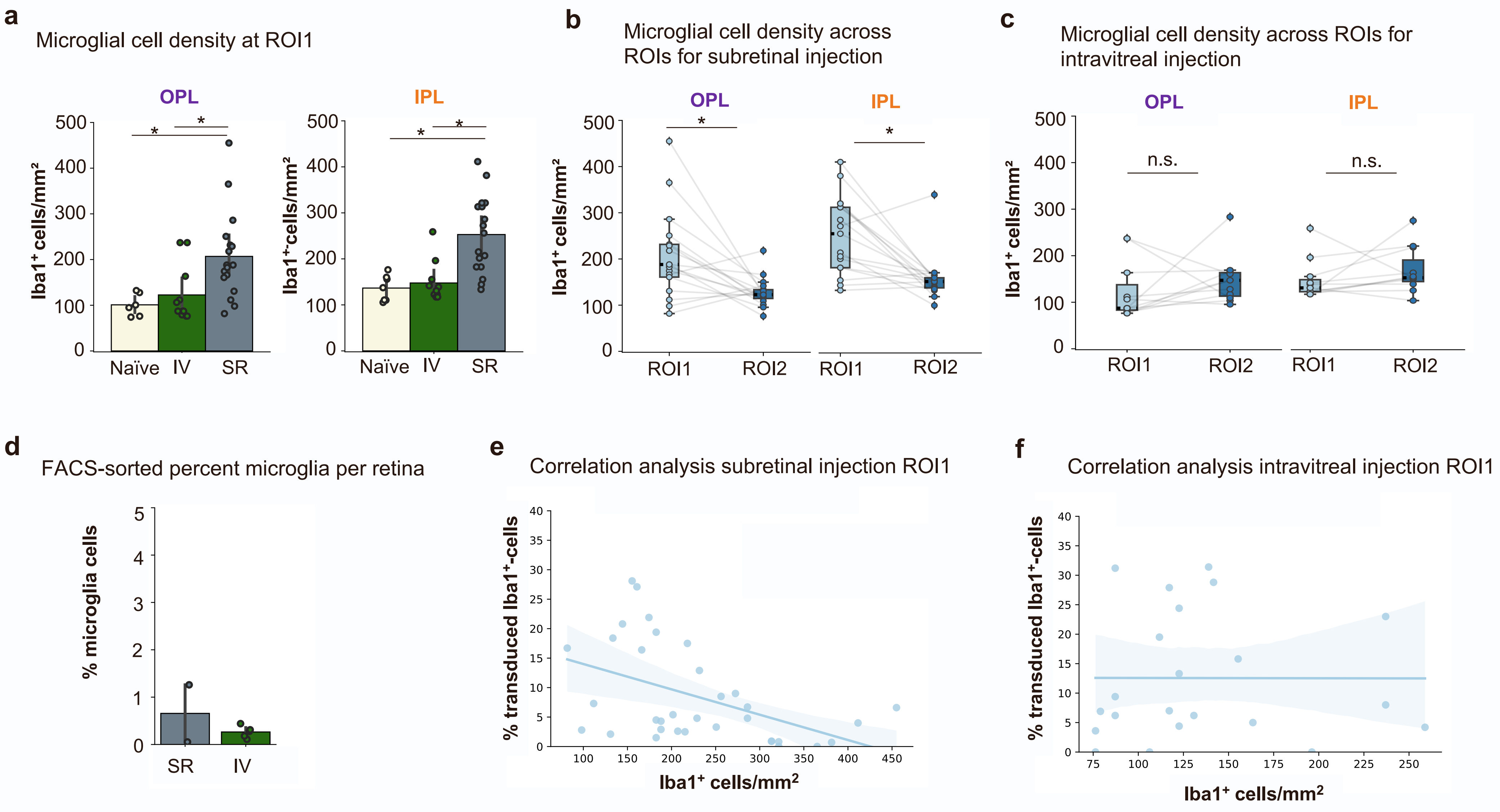

Figure S2. Microglial cell density after viral injection

(**a**) Quantification of $OPL_{microglia}$ and $IPL_{microglia}$ cell densities per mm^2 in retinas from naïve (un-injected), intravitreal or subretinal injections at ROI1. (Kruskal-wallis test: OPL, P = 0.0002; IPL, P = 0.003; post-hoc comparisons Wilcoxon rank-sum test: OPL SR-Naïve, P = 0.002; OPL SR-IV, P = 0.0004; IPL SR-Naïve, P = 0.004; IPL SR-IV, P = 0.010). (**b, c**) Comparison of microglial density between ROIs for both OPL and IPL niche after subretinal (Wilcoxon signed-rank test: OPL, P = 0.004; *IPL*, P = 0.004) or intravitreal injections (**c,** Wilcoxon signed-rank test: OPL, P = 0.248; *IPL*, P = 0.153). (**b**) Percent of microglia ($CD11b^{hi}/CD45^{lo}$) in total cell population of FACS-sorted IV or SR injected retinas. (**e, f**) Correlation using linear regression model between microglial cell density and transduction efficiency for subretinal (*Pearson's r* = -0.476, P = 0.004) or (**e**) intravitreal injection (*Pearson's r* = 0.002, P = 0.992). *$P < 0.05$, ${}^{ns}P >$ 0.05. OPL, outer plexiform layer; IPL, inner plexiform layer; ROI, region of interest. Iba1, ionized calcium binding adaptor molecule 1; SR, subretinal; IV, intravitreal; n.s., not significant.

Figure S3

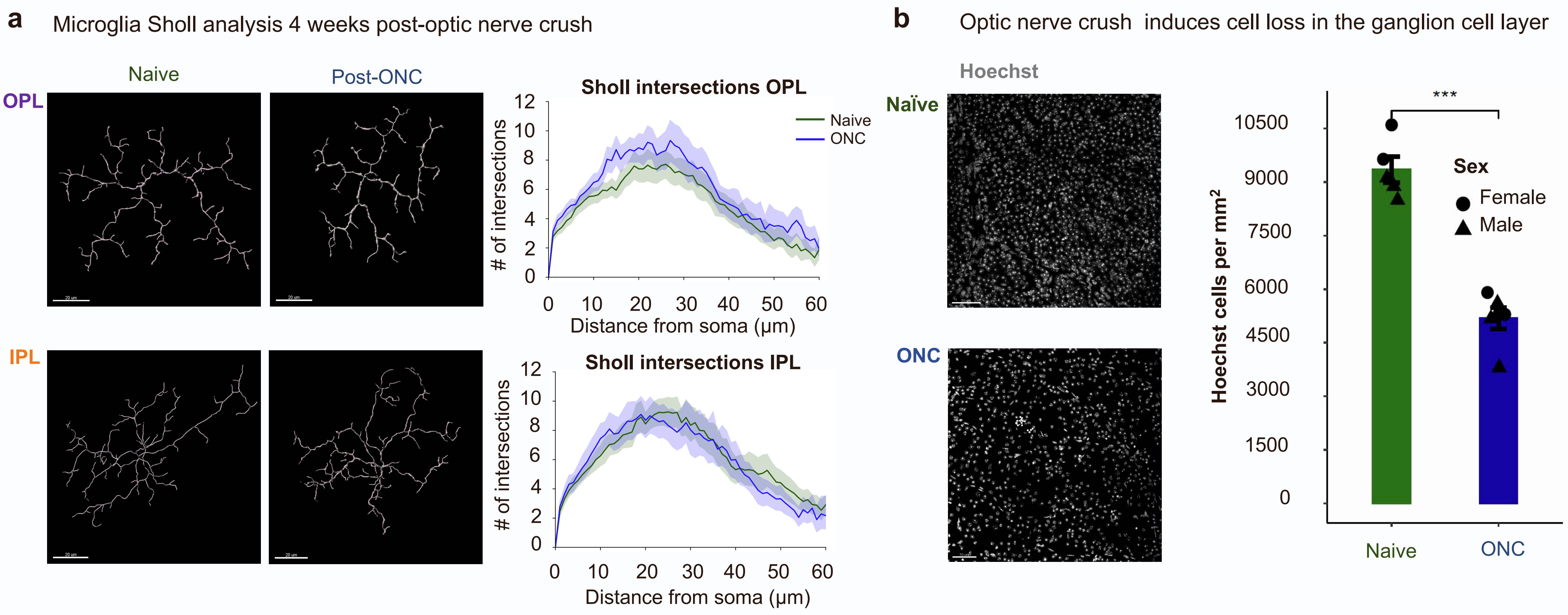

Figure S3. Microglial morphology and ganglion cell loss four weeks after optic nerve crush
(**a**) Example microglial tracings for naïve or four weeks post-optic nerve crush in OPL or IPL. Sholl intersections quantification from 40x 2x2 tiled images from >18 cells from at least 2 animals for each layer and condition. Scale bar: 20µm. (**b**) Hoechst-stained retinal whole mount images of ganglion cell layer (GCL). Total number of nuclei in the GCL is reduced in ONC. Scale bar: 50µm (Welch's Two-sample t-test: *P* = 0.00001). ***P< 0.005. OPL, outer plexiform layer; IPL, inner plexiform layer; ONC, optic nerve crush;

Figure S4

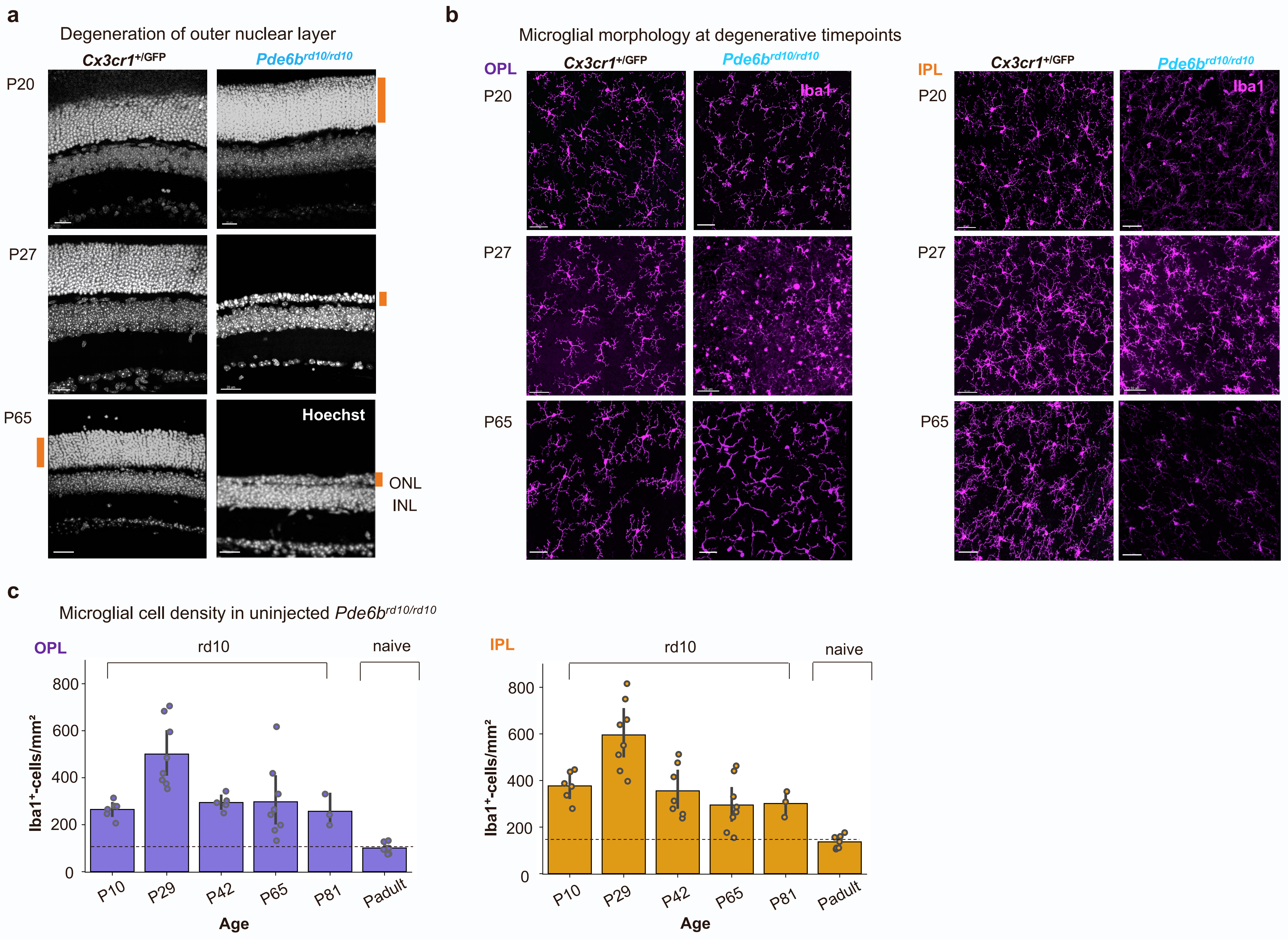

Figure S4. ONL thinning and microglial phenotype in *Pde6b^rd10/rd10* degeneration model
(**a**) Hoechst-labeled retinal cross sections from control *Cx3cr1*$^{GFP/+}$ or *Pde6b*$^{rd10/rd10}$ mice at P20, P27 and P65. Orange bar highlights ONL thickness. Scale bar: 20µm. (**b**) Retinal wholemounts immunostained with Iba1 (magenta) depicting $OPL_{microglia}$ or $IPL_{microglia}$ in control *Cx3cr1*$^{GFP/+}$ or *Pde6b*$^{rd10/rd10}$ mice. Scale bar: 50µm. (**c**) Microglial cell density in OPL or IPL of uninjected *Pde6b*$^{rd10/rd10}$ compared to naïve (dashed line) adult mice. (**d**) Cell density at ROI1 of OPL or IPL microglia in P65 *Pde6b*$^{rd10/rd10}$ retinas injected with scAAV2/6^{TYY}-CD68-eGFP. Cx3cr1, CX3C chemokine receptor 1; ONL, outer nuclear layer; INL, inner nuclear layer. P, postnatal day; Iba1, ionized calcium binding adaptor molecule 1; OPL, outer plexiform layer; IPL, inner plexiform layer; eGFP, enhanced green fluorescent protein; *CD68*, Cluster of differentiation 68.

Figure S5

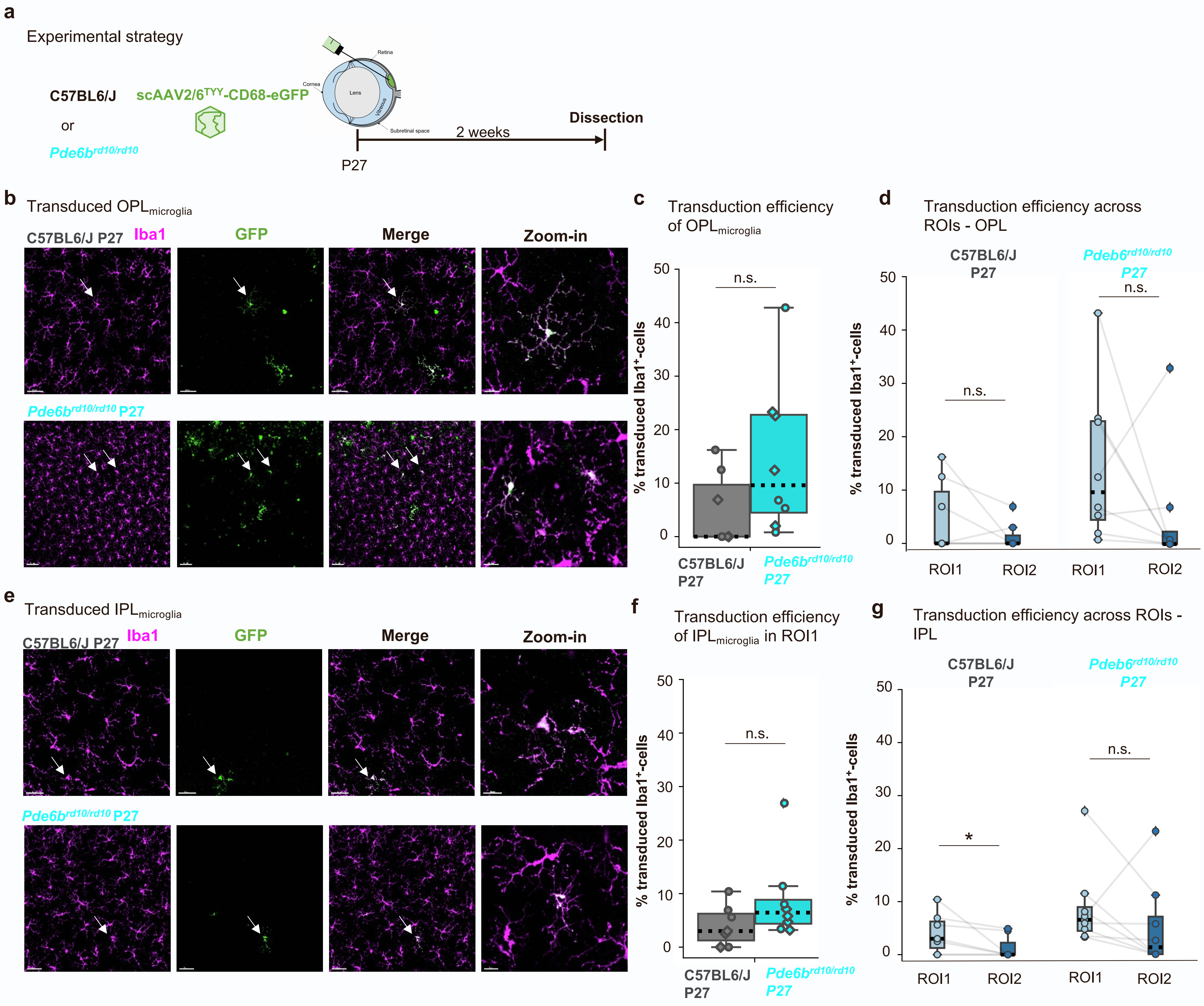

Figure S5. ONL loss in P27 *Pde6b$^{rd10/rd10}$* benefits OPL microglia

(**a**) Experimental timeline. *Pde6b$^{rd10/rd10}$* or C57BL6/J mice received subretinal injection of scAAV2/6^{TYY}-CD68-eGFP (1.37*10^{11}gc/mL) at postnatal day 27 (P27) and retinas were collected 2 weeks after injection. (**b, e**) Retinal wholemounts of OPL$_{microglia}$ (**b**) and IPL$_{microglia}$ (**e**) from indicated strain after subretinal injection stained with Iba1 (magenta) and eGFP (green). White arrows indicate zoom-in region. Scale bar: 50µm, zoom-in: 15µm. (**c, f**) Percent transduction efficiency in OPL$_{microglia}$ (**c,** Wilcoxon ranked-sum test, *P*=0.105) and IPL$_{microglia}$ (**f,** Wilcoxon ranked-sum test, *P*=0.132) Stats. (**d, g**) Transduction across ROIs in C57BL6/J and P27 *Pde6b$^{rd10/rd10}$* retinas in the OPL (**d,** Wilcoxon signed-rank test: C57BL6/J, *P* = 0.144; *Pde6b$^{rd10/rd10}$*, *P* = 0.123) and IPL (**g,** Wilcoxon signed-rank test: C57BL6/J, *P* = 0.043; *Pde6b$^{rd10/rd10}$*, *P* = 0.161). *Pde6b$^{rd10/rd10}$*: n= 8 retinas, 7 mice. C57BL6/J: n= 7 retinas, 4 mice. *$P < 0.05$, $^{ns}P > 0.05$. scAAV, self-complementary adeno-associated virus; P, postnatal day; Iba1, ionized calcium binding adaptor molecule 1; GFP, green fluorescent protein; eGFP, enhanced green fluorescent protein; *CD68*, Cluster of differentiation 68; OPL, outer plexiform layer; IPL, inner plexiform layer; ROI, region of interest; n.s., not significant.

Figure S6

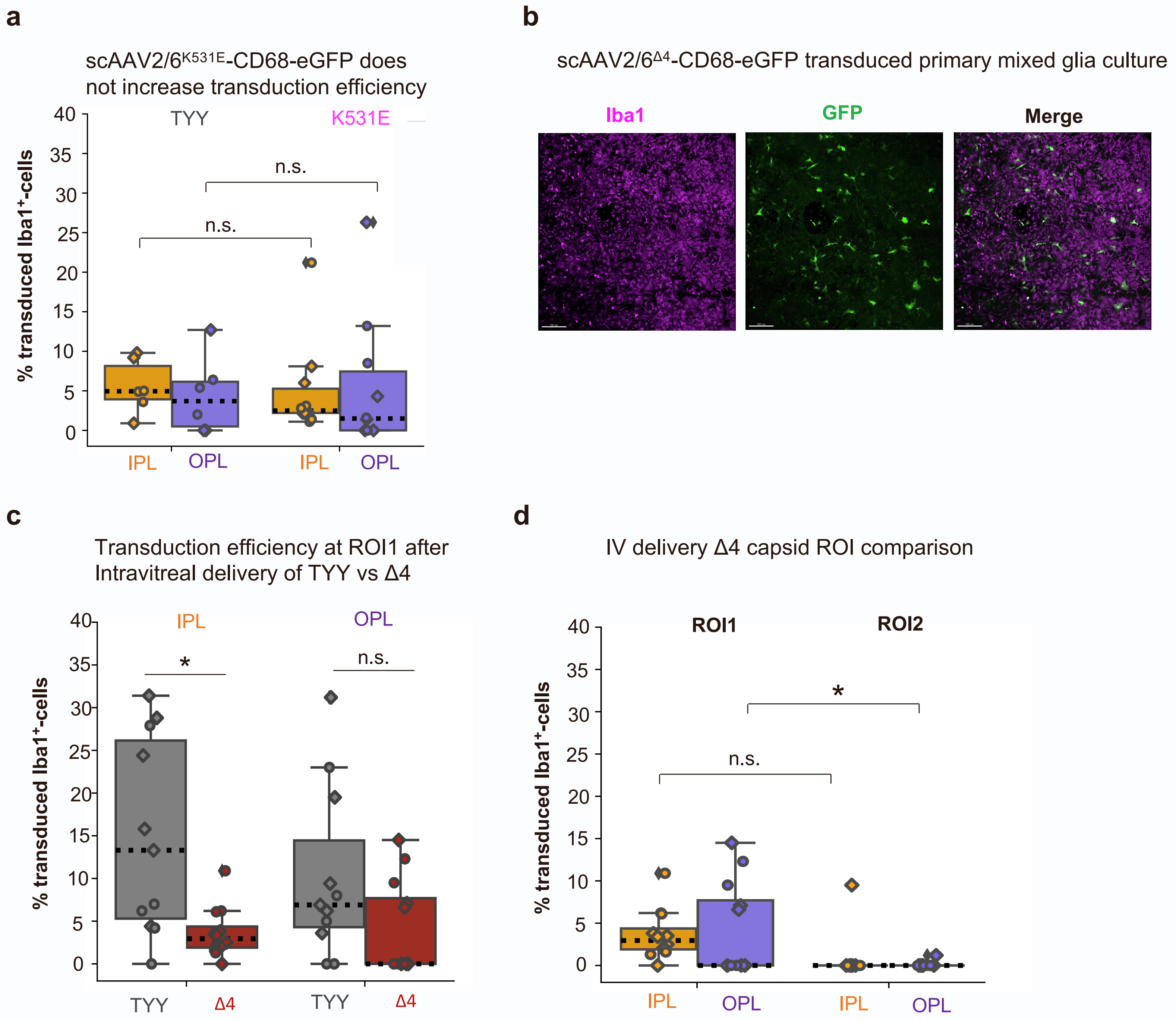

Figure S6. Single heparin-binding mutant and intravitreal delivery of AAV2/6 $^{\Delta4}$
(**a**) Single capsid mutant scAAV2/6^{K531E}-CD68-eGFP microglial transduction efficiency compared to scAAV2/6^{TYY}-CD68-eGFP (Wilcoxon ranked-sum test, OPL, $P = 0.828$; IPL, $P = 0.329$). (**b**) Mixed primary glia cultures transduced with 1*10^{8} viral genomes/well of scAAV2/6$^{\Delta4}$-CD68-eGFP counterstained with Iba1 (magenta). Scale bar: 200 µm. (**c**) Comparison of intravitreally delivered scAAV2/6^{TYY}-CD68-eGFP and scAAV2/6$^{\Delta4}$-CD68-eGFP transduction efficiency of $OPL_{microglia}$ and $IPL_{microglia}$ (Wilcoxon ranked-sum test: OPL, $P = 0.139$; IPL, P=0.005). (**d**) Comparison across ROIs for both OPL and IPL niche after intravitreal delivery of scAAV2/6$^{\Delta4}$-CD68-eGFP. TYY: 11 retinas, 6 mice. Δ4: 12 retinas, 6 mice. *$P < 0.05$, $^{ns}P > 0.05$. scAAV, self-complementary adeno-associated virus; *CD68*, Cluster of differentiation 68; Iba1, ionized calcium binding adaptor molecule 1; IPL, inner plexiform layer; OPL, outer plexiform layer; GFP, green fluorescent protein; ROI, region of interest; TYY, scAAV2/6^{TYY}; Δ4, scAAV2/6$^{\Delta4}$; IV, intravitreal; n.s., not significant.

Figure S7

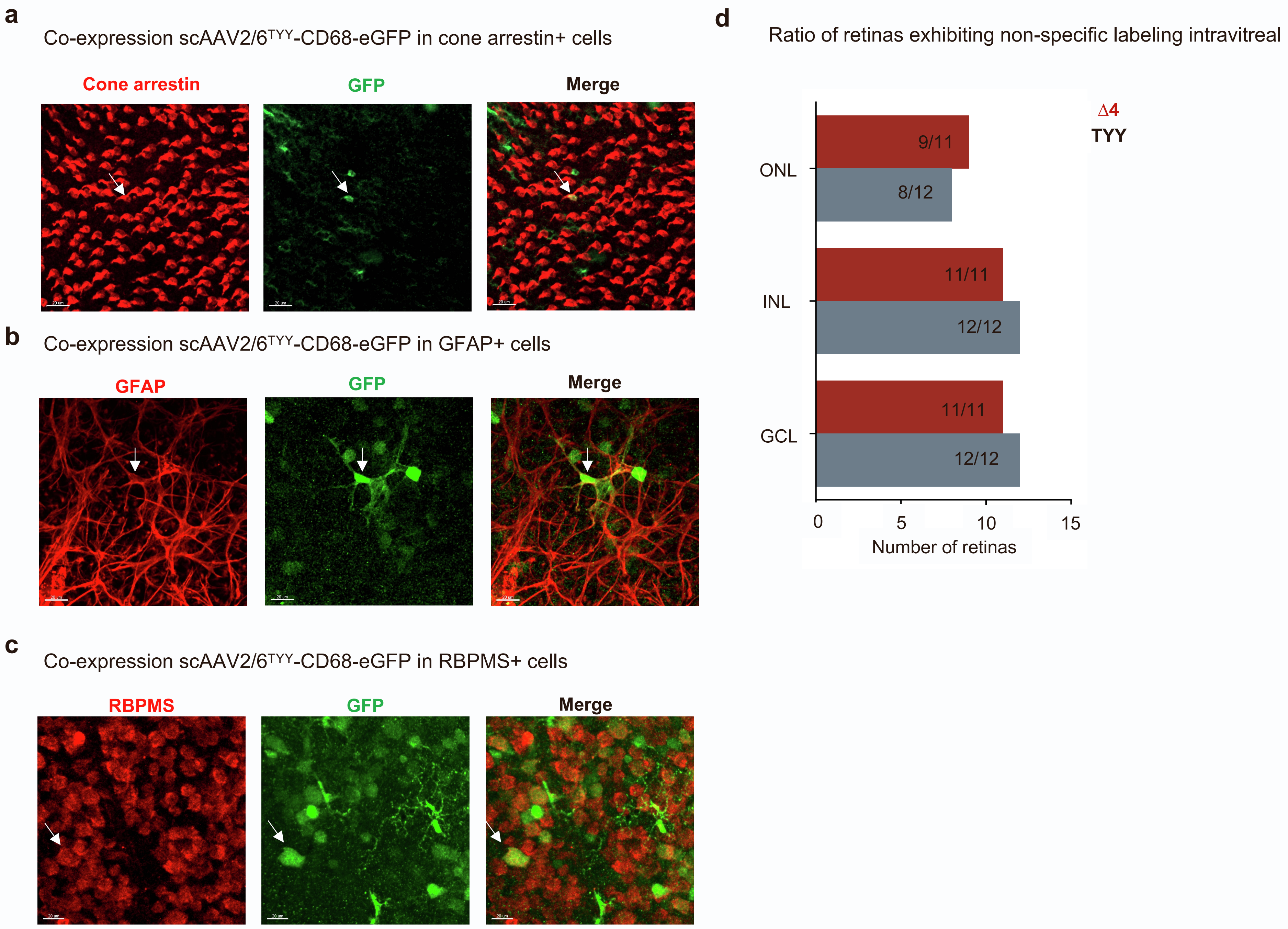

Figure S7. Non-specific labeling using scAAV2/6^{TYY}-CD68-eGFP

Retinal wholemounts of (**a**) cone photoreceptors (cone arrestin^{+}, red), (**b**) astrocytes (GFAP^{+}, red), (**c**) retinal ganglion cells (RPBMS^{+}) co-expressing eGFP (green) following subretinal injection of scAAV2/6^{TYY}-CD68-eGFP (1*10^{12} gc/mL). White arrow indicates co-localized cell. Scale bar: 50µm. (**d**) Ratio of analyzed retinas exhibiting non-specific eGFP expression after subretinal delivery of scAAV2/6^{TYY}-CD68-eGFP across the nuclear layer, outer nuclear layer (ONL), inner nuclear layer (INL) and ganglion cell layer (GCL). scAAV, self-complementary adeno-associated virus; CD68, Cluster of differentiation 68; eGFP, enhanced green fluorescent protein; GFP, green fluorescent protein; GFAP, Glial Fibrillary Acidic Protein; RBPMS, RNA binding protein with multiple splicing; ONL, outer nuclear layer; INL, inner nuclear layer; GCL, ganglion cell layer; TYY, scAAV2/6^{TYY}; Δ4, scAAV2/6^{Δ4}.

Figure S8

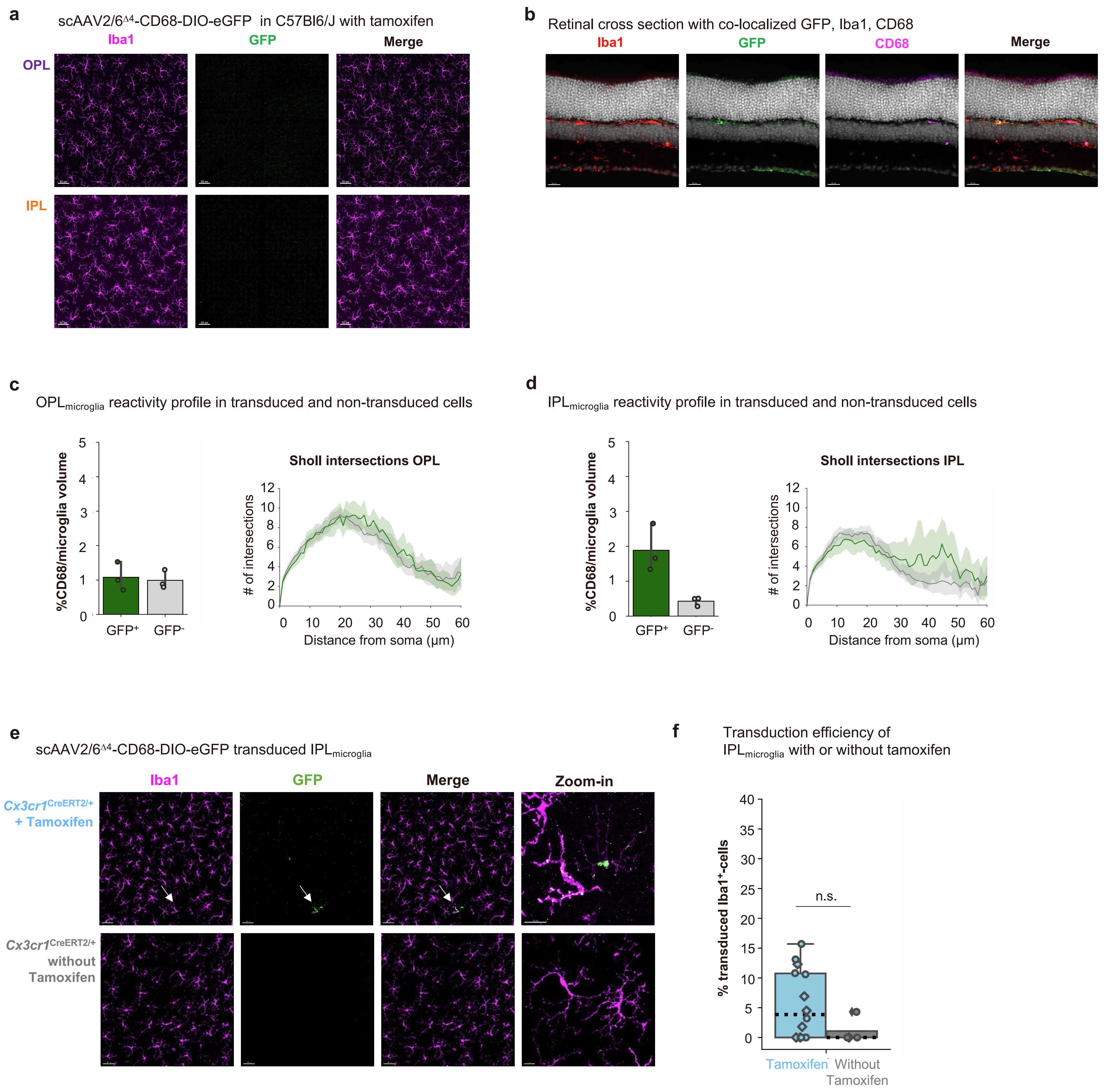

Figure S8. IPL$_{microglia}$ transduction after scAAV$^{\Delta4}$-CD68-DIO-eGFP delivery

(**a**) Retinal wholemounts of OPL and IPL microglia of C57BL6/J mice after subretinal delivery of scAAV$^{\Delta4}$-CD68-DIO-eGFP and tamoxifen treatment immunostained with Iba1 (magenta) and eGFP (green). Scale bar: 50µm. (**b**) Retinal cross section of scAAV2/6$^{\Delta4}$-CD68-DIO-eGFP injected in a *Cx3cr1*$^{CreERT2/+}$ mouse demonstrating colocalization of Iba1 (red), eGFP (green), CD68 (magenta). Nuclei stained with Hoechst (white). Scale bar: 20µm. (**c**) Quantification of % CD68 per microglia volume and Sholl analysis of cell morphology in transduced or untransduced OPL$_{microglia}$ and (**d**) IPL$_{microglia}$. (OPL: n = 47 cells non-transduced, n=15 cells transduced; 3 retinas; IPL: n = 44 cells non-transduced, n=15 cells transduced; 4 retinas) (**e**) Retinal wholemounts of in *Cx3cr1*$^{CreERT2/+}$ mice after subretinal delivery of scAAV$^{\Delta4}$-CD68-DIO-eGFP with or without tamoxifen induction immunostained with Iba1 (magenta) and eGFP (green). White arrows indicate zoom-in. Scale bar: 50µm, zoom-in: 15µm. (**e**) Comparison of transduction efficiency in the IPL with and without tamoxifen (Wilcoxon ranked-sum test: $P = 0.151$). C57BL6/J: 12 retinas, 7 mice. *Cx3cr1*$^{CreERT2/+}$ with Tam: 14 retinas, 9 mice. *Cx3cr1*$^{CreERT2/+}$ without Tam: 4 retinas, 3 mice. $^*P < 0.05$, $^{ns}P > 0.05$. scAAV, self-complementary adeno-associated virus; *CD68*, Cluster of differentiation 68; GFP, green fluorescent protein; eGFP, enhanced green fluorescent protein; Iba1, ionized calcium binding adaptor molecule 1; IPL, inner plexiform layer; OPL, outer plexiform layer; Cx3cr1, CX3C chemokine receptor 1; DIO, double-floxed inverse orientation; CreERT2, tamoxifen-inducible Cre recombinase; Tam, Tamoxifen; n.s., not significant.
