# Supplementary material for: Optimizing AAV2/6 microglial targeting identified enhanced efficiency in the photoreceptor degenerative environment
Source: Mol Ther Methods Clin Dev. 2021 Sep 14;23:210–24. doi: 10.1016/j.omtm.2021.09.006 (PMC8516996; doi:10.1016/j.omtm.2021.09.006)
Supplement: Document S2. Article plus supplemental information [file mmc2.pdf]

Molecular Therapy
Methods & Clinical Development
Original Article

Optimizing AAV2/6 microglial targeting identified enhanced efficiency in the photoreceptor degenerative environment

Margaret E. Maes,[1,3] Gabriele M. Wögenstein,[1,2,3] Gloria Colombo,[1] Raquel Casado-Polanco,[1] and Sandra Siegert[1]

[1]Institute of Science and Technology (IST) Austria, 3400 Klosterneuburg, Austria

Adeno-associated viruses (AAVs) are widely used to deliver genetic material *in vivo* to distinct cell types such as neurons or glial cells, allowing for targeted manipulation. Transduction of microglia is mostly excluded from this strategy, likely due to the cells' heterogeneous state upon environmental changes, which makes AAV design challenging. Here, we established the retina as a model system for microglial AAV validation and optimization. First, we show that AAV2/6 transduced microglia in both synaptic layers, where layer preference corresponds to the intravitreal or subretinal delivery method. Surprisingly, we observed significantly enhanced microglial transduction during photoreceptor degeneration. Thus, we modified the AAV6 capsid to reduce heparin binding by introducing four point mutations (K531E, R576Q, K493S, and K459S), resulting in increased microglial transduction in the outer plexiform layer. Finally, to improve microglial-specific transduction, we validated a Cre-dependent transgene delivery cassette for use in combination with the *Cx3cr1*$^{\text{CreERT2}}$ mouse line. Together, our results provide a foundation for future studies optimizing AAV-mediated microglia transduction and highlight that environmental conditions influence microglial transduction efficiency.

INTRODUCTION

Viral vector engineering has become an effective strategy for *in vivo* delivery of genetic material to distinct cell populations. Due to their ease of engineering and production,[1] adeno-associated viruses (AAVs) are widely used to target various cell types of the central nervous system (CNS).[2] However, microglia, the resident immune cells of the CNS, are excluded from this success. Microglial transduction *in vivo* is only occasionally reported,[3–5] and its overall low efficiency limits microglial manipulation *in vivo*. Yet, we need strategies to selectively alter microglia to obtain knowledge about their function within local environments and to identify their impact in disease onset and progression.[6,7] Currently, the field is limited to generating gene-of-interest knockout mouse models[8,9] or to a small number of microglia receptor-specific inhibitors (e.g., for activity alteration [purinergic receptor P2Y12] or depletion [colony-stimulating factor 1 receptor] strategies).[10,11] Therefore, AAVs could provide a comparatively quick alternative to deliver the desired cargo necessary to investigate new research questions. So far, the lack of robust and systematic investigation of *in vivo* targeting strategies has hindered viral delivery optimization for microglia and is a knowledge gap that needs to be addressed.

Successful viral transduction strategies depend on maximizing cell-specific targeting and minimizing off-target gene expression. By combining known cellular tropism of AAV capsid serotypes with cell-type-selective promoters, this goal has been met for many neurons and glia.[12,13] For microglia, neither *in vitro* screens of several capsids with a constitutive promoter[14,15] nor an *in vivo* screen of three capsids and over 200 synthetic promoters[14,16] led to sufficient or reportable microglia transduction.[5] So far, AAV2/6^{TYY} is the most encouraging AAV for targeting microglia because it contains capsid mutations that prevent proteasomal degradation upon target cell entry.[17] This AAV2/6^{TYY} was reported to target hippocampal microglia *in vivo*,[14] although the *in vitro* transduction efficiency was much higher. This discrepancy could be explained by the difference between *in vitro* and *in vivo* microglial transcriptional signature.[18–20] Therefore, optimization of AAV to transduce microglia should be performed in an *in vivo* setting, which requires an anatomically defined and well-controlled environment. The retina provides an ideal model, as its highly ordered structure demarcates two synaptic layers, each occupied by distinct microglial niches.[21,22] Viral delivery is fast and minimally invasive, and several well-characterized degenerative disease models are available, along with known disease-associated microglial genes.[6]

Here, we first assessed the feasibility for *in vivo* AAV-mediated microglial transduction in the retina using scAAV2/6^{TYY}-CD68-eGFP. We systematically investigated different viral delivery strategies and whether a degenerative environment affected microglia susceptibility to AAV transduction. Surprisingly, microglial transduction and viral spread improved across the plexiform layers and laterally through the retina when degeneration was initiated in the outer retinal layer.

Received 17 May 2021; accepted 7 September 2021;
https://doi.org/10.1016/j.omtm.2021.09.006.

[2]Present address: University Children's Hospital Zurich, University of Zurich (UZH), 8032 Zurich, Switzerland

[3]These authors contributed equally

Correspondence: Sandra Siegert, Institute of Science and Technology (IST) Austria, 3400 Klosterneuburg, Austria.
E-mail: sandra.siegert@ist.ac.at

This is an open access article under the CC BY license (http://creativecommons.org/licenses/by/4.0/).

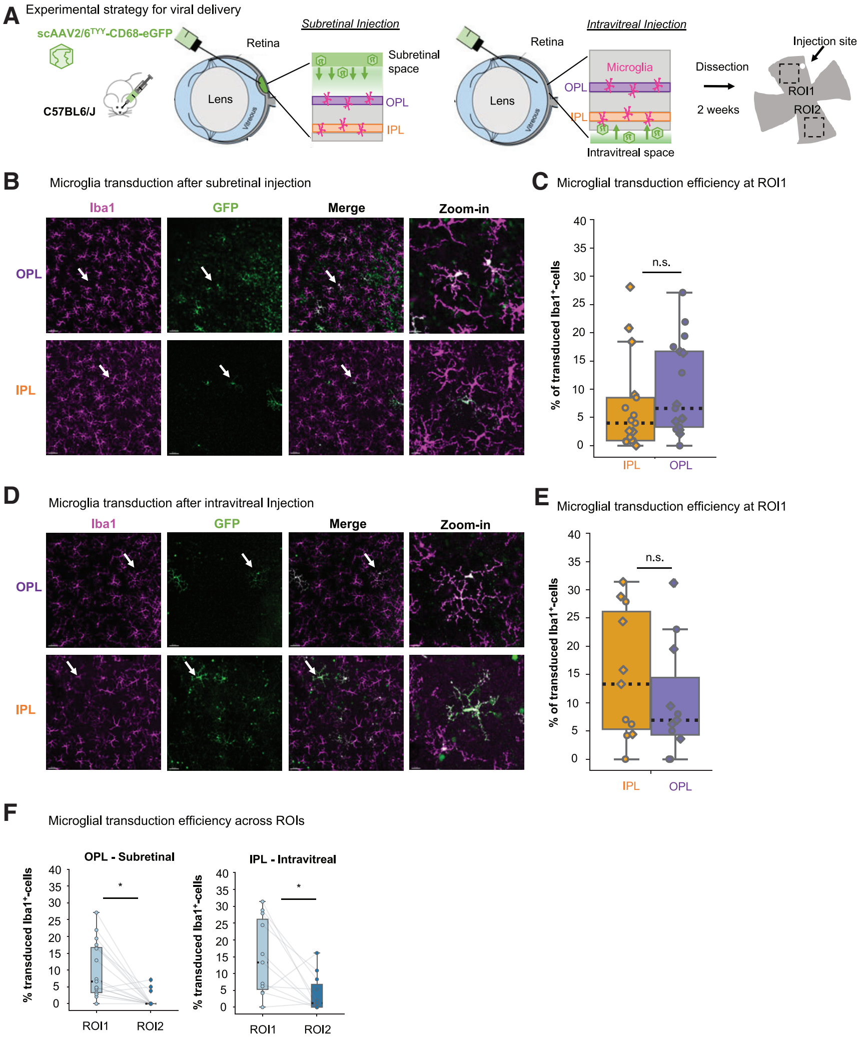

Figure 1. Viral delivery route influences preferred microglial layer transduction

(A) Experimental strategy. Adult C57BL6/J mice injected with scAAV2/6^{TYY}-CD68-eGFP (1×10^{12} gc/mL) through subretinal or intravitreal delivery route and collected 2 weeks later. Images acquired in ROI1, defined as the quadrant closest to the injection site, and ROI2, the opposing quadrant. (B and C) Subretinal, (D and E) intravitreal injection. (B and D) Retinal whole-mount images of OPL and IPL after subretinal (B) and intravitreal (D) injection stained with Iba1 (magenta) and eGFP (green). White arrows indicate zoom-in region. Scale bar: 50 μm; zoom-in: 15 μm. (C and E) Percent microglial transduction efficiency (Iba1/eGFP-double-positive/total Iba1^{+} cell numbers) for OPL and IPL microglia at ROI1 after subretinal (C, Wilcoxon signed-rank test: p = 0.246) and intravitreal (E, Wilcoxon signed-rank test: p = 0.286) injection. Each point represents ROI1 from one retina. Diamond: male; circle: female. (F) Comparison of transduction efficiency across ROIs for individual retinas analyzed in OPL after subretinal (Wilcoxon signed-rank test: p = 0.001) or IPL after intravitreal injection (Wilcoxon signed-rank test: p = 0.021). Gray lines connect ROIs from a single retina. Subretinal: 17 retinas, 9 mice. Intravitreal: 11 retinas, 6 mice. *$p < 0.05$, $^{ns}p > 0.05$. scAAV, self-complementary adeno-associated virus; CD68, cluster of differentiation 68; eGFP, enhanced green fluorescent protein; gc/mL, genome copies per milliliter; Iba1, ionized calcium-binding adaptor molecule 1; IPL, inner plexiform layer; n.s., not significant; OPL, outer plexiform layer; ROI, region of interest.

Based on this finding, we engineered an AAV capsid to promote spread within the outer retina in a non-degenerative environment by introducing heparin-binding mutations to the AAV6 capsid. We confirmed that this new AAV2/6 capsid led to selective transduction in microglia of the outer plexiform layer (OPL). Finally, we optimized the AAV specificity to reduce off-target expression in other retinal cells by combining a double-inverted transgene cassette with the *Cx3cr1*^{CreERT2} (CX3C chemokine receptor 1) mouse line. Overall, this work established the retina as a model system to validate future AAV modification for microglial transduction, which will be relevant for application in other brain regions.

RESULTS

Viral delivery route corresponds with layer-specific microglial transduction and viral spread

To investigate whether retinal microglia can be successfully transduced with AAV, we took advantage of scAAV2/6^{TYY}-CD68-eGFP, which consists of a modified AAV6^{TYY} capsid and a transgene encoding for enhanced green fluorescent protein (eGFP) driven under the monocyte and tissue macrophage-selective cluster of differentiation 68 (*CD68*) promoter (Figure S1A).[14] *CD68* transcripts are reliably found *in vivo* in brain microglia,[23] as well as in the adult retina.[6] Furthermore, a 5′ mutated inverted terminal repeat (ITR) flanks the AAV2 genome for self-complementary (sc) assembly and faster transgene expression.[14] After scAAV2/6^{TYY}-CD68-eGFP production, we confirmed eGFP expression in microglia of primary mixed glial culture *in vitro* (Figure S1B).

Classically in rodent studies, the location of the retinal cell type to be targeted dictates which injection strategy will be used, where subretinal or intravitreal injection preferentially transduces cells in the outer or inner retinal layers, respectively (Figure 1A).[24–26] Microglia in the adult retina are localized in both the outer and inner plexiform layer (OPL and IPL, respectively; Figure S1C). Thus, to determine which injection method robustly targets OPL$_{microglia}$ and/or IPL$_{microglia}$, we subretinally or intravitreally injected scAAV2/6^{TYY}-CD68-eGFP into adult C57BL6/J mice (Figure 1A). After 2 weeks, we performed immunostaining for eGFP and ionized calcium-binding adaptor molecule 1 (Iba1) to label the microglial population.[27] To assess microglial transduction efficiency, we calculated the ratio of

eGFP$^+$/Iba1$^+$ cells to the total number of Iba1$^+$ cells within region-of-interest 1 (ROI1), defined as the retinal quadrant closest to the injection site (Figure 1A). The median microglial transduction efficiency for both plexiform layers was 6.6% for subretinal and 13% for intravitreal injection, with eGFP frequently expressed in non-microglia cells (Figures S1D and S1E). We also assessed the overall transduction efficiency using flow cytometry (Figure S1F) and found that 1.5% of total cells were eGFP$^+$ independent from the injection method, while less than 10% of the 1.5% eGFP$^+$ population was microglia (Figures S1G and S1H). When we quantified the microglial transduction efficiency of only OPL or IPL, subretinal injection resulted in higher efficiency of $OPL_{microglia}$ compared to $IPL_{microglia}$ (Figures 1B and 1C), and vice versa for intravitreal injection (Figures 1D and 1E). This relationship held true when comparing $OPL_{microglia}$ or $IPL_{microglia}$ efficiencies across injection methods (Figure S1I). To estimate the viral spread throughout the retina, we analyzed the opposing quadrant from the injection site, region-of-interest 2 (ROI2; Figure 1A). Independent from the injection method, the transduction efficiency was significantly reduced between ROI1 and ROI2 (Figure 1F). However, intravitreal injection maintained a slightly higher transduction level for ROI2, suggesting enhanced viral spread. Although quantification at two ROIs allowed for assessment of layer-specific transduction and viral spread, with this method, we can only estimate whole-retina microglial transduction efficiency. Using flow cytometry, we assessed the percentage of eGFP$^+$ CD11b^{hi}CD45^{lo} cells and detected a mean transduction efficiency of 45% and 25% for intravitreal and subretinal injection (Figure S1J).

Since both viral delivery strategies caused minor injury, thereby initiating microglial proliferation,[28,29] we compared the microglial density within ROI1 to naive, non-injected animals (Figure S2A). Only the subretinal method increased microglial density in both plexiform layers. When we assessed each ROI separately, the effect only occurred in ROI1, whereas ROI2 remained at the naive level (Figure S2B). Intravitreal injection did not affect microglial cell density (Figure S2C). We detected a similar relationship in whole-retina analysis by flow cytometry (Figure S2D). To confirm that increased efficiency at ROI1 is not due to increased cell density, we calculated the Pearson's coefficient for both subretinal and intravitreal injections at ROI1 (Figures S2E and S2F). We found a negative correlation for subretinal injection, suggesting we may underestimate efficiency, while there was no effect for intravitreal injection.

Together, our data show that scAAV2/6^{TYY}-CD68-eGFP successfully transduced retinal microglia, preferentially at the ROI closest to the injection site, and that the viral delivery route influences layer preference and viral diffusion across the microglial population.

Loss of inner retinal barriers did not improve microglial transduction after intravitreal delivery

Effective viral-mediated transgene delivery faces multiple challenges *in vivo*, like physical barriers.[30] Intravitreally delivered viral particles must first bypass the inner limiting membrane, the dense extracellular matrix around the nerve fiber, and the ganglion cell layer to reach the IPL.[31] Disrupting these barriers using the optic nerve crush (ONC) model has been shown to result in greater penetration into the retinal layers for AAV2.[32–34] To determine whether ONC influenced microglial transduction, we performed ONC on adult C57BL6/J mice and intravitreally injected scAAV2/6^{TYY}-CD68-eGFP 4 weeks post-injury (Figure 2A). At this time, microglia have cleared the apoptotic ganglion cells[35] and returned to a non-reactive morphology, as confirmed by Sholl analysis (Figure S3A). Two weeks after the injection, we analyzed the retinas for eGFP$^+$ microglia and compared to non-crushed, intravitreally injected retinas. Unexpectedly, neither the $OPL_{microglia}$ nor the $IPL_{microglia}$ transduction efficiency improved (Figures 2B–2E), even though ONC resulted in a 50% cell loss in the ganglion cell layer (Figure S3B). The viral spread was also unchanged in the IPL (Figure 2F). Thus, the ONC-mediated reduction of the physical barrier did not further improve microglial transduction.

Rod photoreceptor loss enhanced microglial transduction after subretinal delivery

Subretinally delivered viral particles must pass the densely packed outer nuclear layer (ONL) to target cells in the OPL. Photoreceptor degeneration models, such as retinal degeneration model 10 (*rd10*), reduce this physical barrier. *Rd10* harbors a missense point mutation in the *Pde6b* gene leading to progressive rod photoreceptor degeneration,[36] which peaks at postnatal day 25 (P25)–P30.[37] At P65, the ONL thickness was reduced (Figure S4A), and microglial density was comparable to naive (Figures S4B and S4C). Therefore, we subretinally injected scAAV2/6^{TYY}-CD68-eGFP in *Pde6b*$^{rd10/rd10}$ mice at P65 and age-matched C57BL6/J controls and analyzed the retina 2 weeks post-injection (Figure 3A). We found enhanced $OPL_{microglia}$ transduction in *Pde6b*$^{rd10/rd10}$ compared to controls (Figures 3B and 3C). Also, the median viral spread significantly improved across ROIs by five-fold (Figure 3D). Although subretinal delivery is not the optimal route for targeting $IPL_{microglia}$ (Figure 1E), we unexpectedly found a significant increase at both ROIs (Figures 3E–3G).

Since ONL loss is apparent by P27 in *Pde6b*$^{rd10/rd10}$ (Figure S4A), we investigated whether enhanced transduction is already evident. Therefore, we injected subretinally scAAV2/6^{TYY}-CD68-eGFP at P27 and collected the retinas 2 weeks post-injection (Figure S5A). For $OPL_{microglia}$, the transduction efficiency increased, but only for ROI1 (Figures S5B–S5D). For $IPL_{microglia}$, we no longer observed increased transduction efficiency (Figures S5E and S5F), and there was no change in spread across ROIs for either plexiform layer (Figure S5G), which is in contrast to *Pde6b*$^{rd10/rd10}$ P65.

Taken together, the loss of the physical ONL barrier in *Pde6b*$^{rd10/rd10}$ benefits $OPL_{microglia}$ transduction efficiency, while the P65 environment further supports transduction across plexiform layers and ROIs.

Mutation of AAV6^{TYY} capsid heparin binding sites improved $OPL_{microglia}$ transduction

Extracellular matrix remodeling in the outer retinal layers could explain the increased transduction and spread in the *Pde6b*$^{rd10/rd10}$ P65 environment. Heparan sulfate proteoglycans (HSPGs), a component of

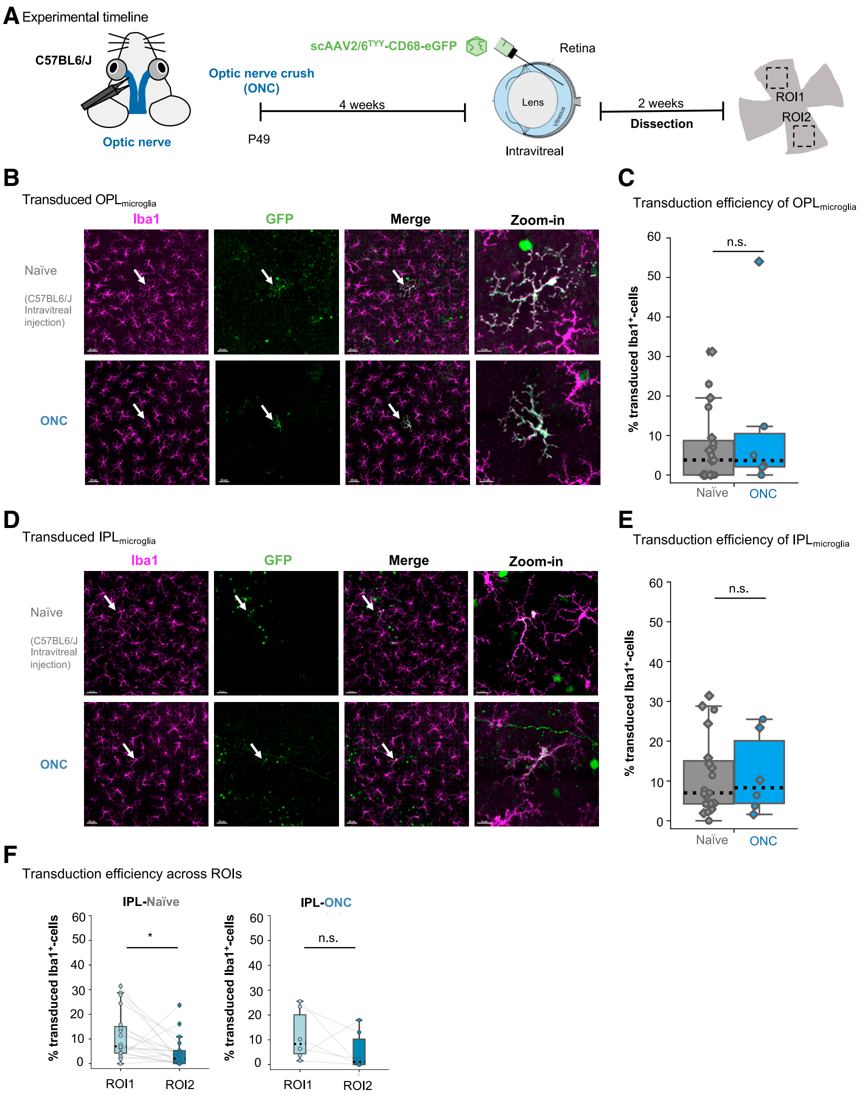

Figure 2. Microglial transduction efficiency was unaltered after optic nerve crush (ONC)

(A) Experimental timeline. ONC surgery performed on the left eye of adult C57BL6/J mice. scAAV2/6^{TYY}-CD68-eGFP intravitreally delivered 4 weeks later. Retinas collected 2 weeks after injection. (B and D) Retinal whole-mount images of OPL$_{microglia}$ or IPL$_{microglia}$ after ONC or naive non-crushed controls stained with Iba1 (magenta) and eGFP (green). White arrows indicate zoom-in region. Scale bar: 50 μm; zoom-in: 15 μm. (C and E) Percent microglial transduction efficiency (Iba1/eGFP-double-positive/total Iba1$^+$ cell numbers) for OPL$_{microglia}$ (C, Wilcoxon rank-sum test: $p = 0.633$) and IPL$_{microglia}$ (E, Wilcoxon rank-sum test: $p = 0.899$) naive non-crushed control or ONC at ROI1. Each point represents ROI1 from one retina. Diamond: male; circle: female. Two experiments pooled (1×10^{12} gc/mL or 1.37×10^{11} gc/mL). (F) Comparison of transduction efficiency across ROIs for individual retinas analyzed in IPL$_{microglia}$ in naive non-crushed control (Wilcoxon signed-rank test: $p = 0.014$) or ONC condition (Wilcoxon signed-rank test: $p = 0.249$). Gray lines connect ROIs from a single retina. Naive non-crushed control: n = 19 retinas, 12 mice. ONC: n = 6 retinas, 6 mice. $^*p < 0.05$, $^{ns}p > 0.05$.

the extracellular matrix, are one of the primary binding receptors between AAVs and the cell surface.[38,39] Boye et al.[40] have shown improved outer retinal transduction through selective capsid mutations at binding sites of heparin, a highly sulfated form of heparan sulfate. A previously identified single mutation, K531E, reduced heparin binding capacity by AAV6.[41] Therefore, we introduced this mutation to the AAV6^{TYY} capsid (scAAV^{K531E}-CD68-eGFP) and performed subretinal injection in adult C57BL6/J animals. When we analyzed the retinas 2 weeks later, microglial transduction efficiency did not improve in either plexiform layer (Figure S6A). Combined heparin-binding mutations increased viral spread;[40] thus, we included three additional mutations (R576Q, K493S, and K459S; Figure 4A).[42] After confirming that the mutated AAV6 capsid (AAV6$^{\Delta4}$) transduced microglia in primary mixed glial cells *in vitro* (Figure S6B), we subretinally injected scAAV2/6$^{\Delta4}$-CD68-eGFP into adult C57BL6/J mice (Figure 4B). OPL$_{microglia}$ showed a two-fold increase in transduction for ROI1 compared to scAAV2/6^{TYY} (Figures 4C and 4D). The efficiency did not improve for OPL$_{microglia}$ in ROI2 (Figure 4E), or for IPL$_{microglia}$ in either ROI (Figures 4F–4H), suggesting that the AAV2/6$^{\Delta4}$ capsid may have limitations in lateral diffusion through the ONL and/or crossing the inner nuclear layer to reach the IPL$_{microglia}$ niche.

Heparin binding is required for AAV to pass the inner limiting membrane;[43] therefore, to validate our mutant capsid scAAV2/6$^{\Delta4}$-CD68-eGFP, we also performed intravitreal injections. Indeed, the percentage of transduced IPL$_{microglia}$ was significantly reduced (Figure S6C). We observed few eGFP$^+$ microglia close to the ROI1 and none in the ROI2 (Figure S6D), suggesting minimal access from the injection procedure. Overall, the mutated heparin binding sites in our scAAV2/6$^{\Delta4}$-CD68-eGFP improved OPL$_{microglia}$ transduction.

Microglial-specific transduction with Cre recombinase-dependent AAV2/6$^{\Delta4}$

Although we improved efficiency with scAAV2/6$^{\Delta4}$-CD68-eGFP, we still frequently encountered off-target transgene expression in cone arrestin$^+$ photoreceptors in the ONL, glial fibrillary acidic protein$^+$ (GFAP) Müller glia in the inner nuclear (INL), and RNA-binding protein with multiple splicing$^+$ (RBPMS) ganglion cells and GFAP$^+$ astrocytes in the ganglion cell layer (GCL) (Figures S7A–S7C and S1H). When we counted the number of retinas with at least one positive off-target cell, all retinas in INL and GCL exhibited off-target cells, and over half in the ONL (Figure S7D). Since this unspecific expression limits downstream applications and due to the lack of

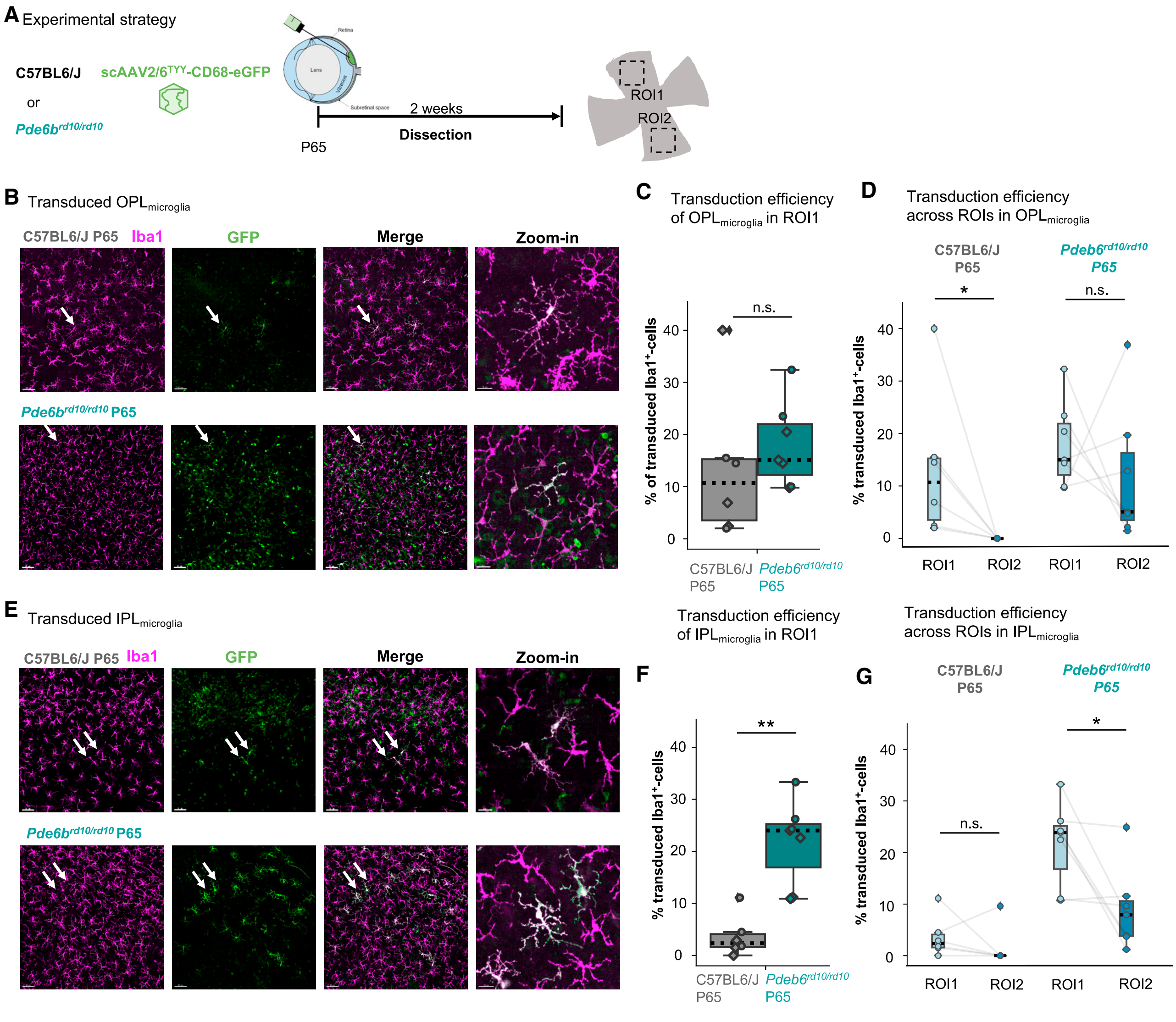

Figure 3. Photoreceptor degeneration increases microglial transduction efficiency and spread throughout retina.
(A) Experimental timeline. Subretinal delivery of scAAV2/6^{TYY}-CD68-eGFP (1.37×10^{11} gc/mL) to postnatal day 65 (P65) *Pde6b*$^{rd10/rd10}$ or P65 C57BL6/J mice. (B and E) Retinal whole mounts of transduced OPL$_{microglia}$ (B) and IPL$_{microglia}$ (E) in P65 *Pde6b*$^{rd10/rd10}$ retinas immunostained with Iba1 (magenta) or eGFP (green). White arrows indicate zoom-in. Scale bar: 50 μm; zoom-in: 15 μm. (C) Comparison of P65 *Pde6b*$^{rd10/rd10}$ and C57BL6/J transduction efficiency (IIba1/eGFP-double-positive/total Iba1^{+} cell numbers) of OPL$_{microglia}$ (Wilcoxon rank-sum test, $p = 0.284$) and (D) transduction across OPL$_{microglia}$ ROIs (Wilcoxon signed-rank test: C57BL6/J, $p = 0.027$; *Pde6b*$^{rd10/rd10}$, $p = 0.398$). (F) IPL$_{microglia}$, transduction efficiency in P65 *Pde6b*$^{rd10/rd10}$ compared to P65 C57BL6/J control (Wilcoxon rank-sum test: $p = 0.004$). (G) Transduction across ROIs in IPL$_{microglia}$ (Wilcoxon signed-rank test: C57BL6/J, $p = 0.345$; *Pde6b*$^{rd10/rd10}$, $p = 0.027$). P65 *Pde6b*$^{rd10/rd10}$: 7 retinas, 4 mice. C57BL6/J: 6 retinas, 4 mice. $^{**}p < 0.005$, $^{*}p < 0.05$, $^{ns}p > 0.05$. *Pde6b*, phosphodiesterase 6B; rd, retinal degeneration.

synthetic microglial-selective promoters,[16] we decided to improve specificity by introducing an inducible double-floxed inverse orientation (DIO) sequence into the transfer vector (Figure 5A). In combination with the *Cx3cr1*^{CreERT2} mouse line,[8] we can selectively induce transgene inversion in microglia upon tamoxifen exposure. This mouse model is advantageous over the constitutive *Cx3cr1*-Cre, since expression of this receptor is only microglia/macrophage specific after embryonic day 18.[44] We subretinally injected the scAAV2/6$^{\Delta 4}$-CD68-DIO-eGFP into *Cx3cr1*$^{CreERT2/+}$ mice. To induce Cre-mediated inversion, we intraperitoneally injected tamoxifen for 3 consecutive days 1 week after viral delivery[45] and analyzed transgene expression 2 weeks later (Figure 5B).

As expected, eGFP expression was selective for microglia and tamoxifen dependent (Figures 5C and 5D). Without tamoxifen, we occasionally observed microglial transduction (Figure 5D), which was expected

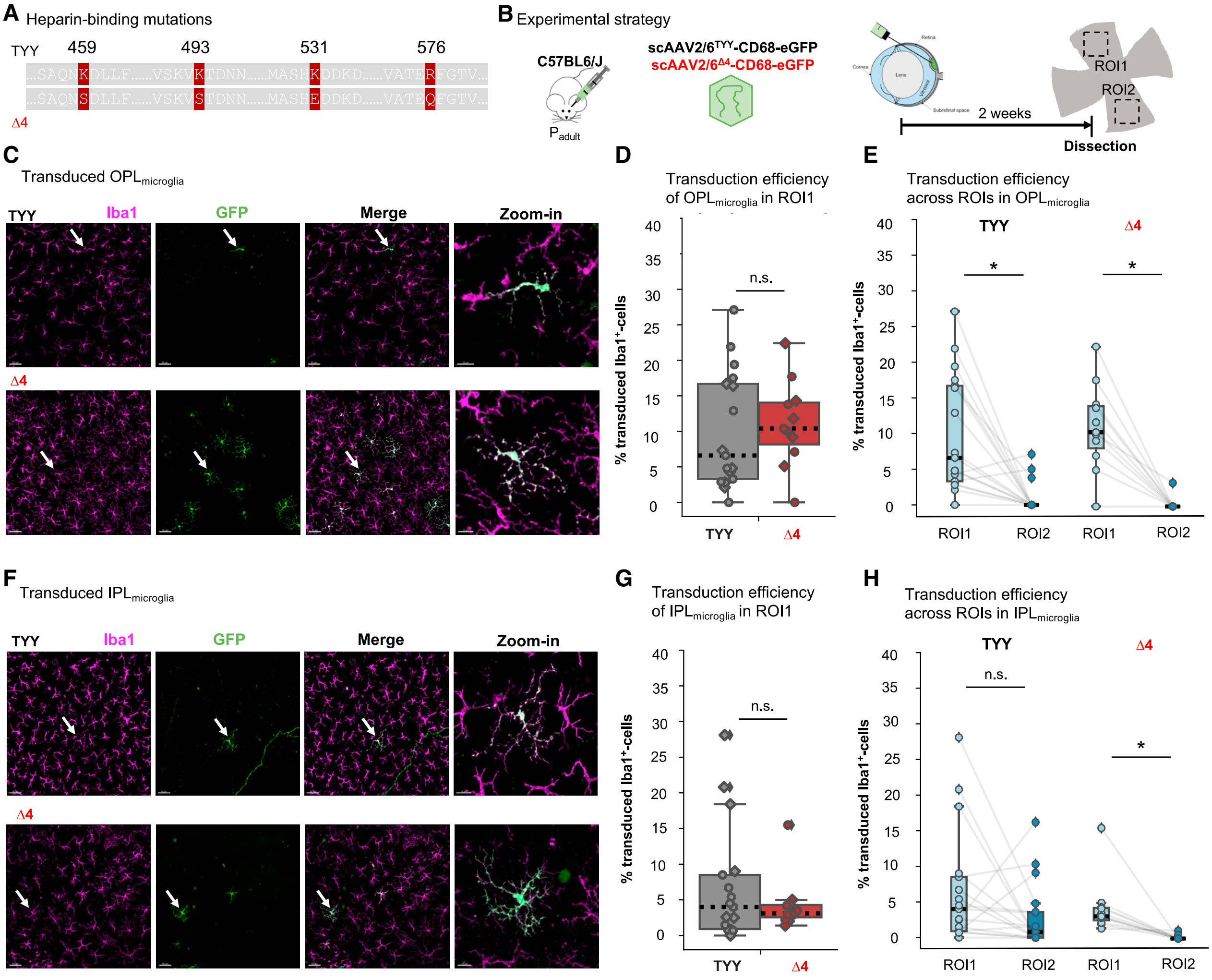

Figure 4. Heparin-binding capsid mutations increase OPL$_{microglia}$ transduction

(A) Depiction of the site-specific mutations in the AAV6 capsid (K459S, K493S, K531E, R576Q). (B) Experimental timeline. Subretinal delivery of scAAV2/6^{TYY}-CD68-eGFP (1×10^{12} gc/mL) or scAAV2/6$^{\Delta4}$-CD68-eGFP (1×10^{12} gc/mL) to adult C57BL6/J mice. Dissection of retinas followed 2 weeks later. (C and F) Retinal whole mounts immunostained with Iba1 (magenta) and eGFP (green) showing transduced OPL$_{microglia}$ and IPL$_{microglia}$ (F) in C57BL6/J retinas (dataset from subretinal Figure 1) with indicated capsid variant. White arrows indicate zoom-in. Scale bar: 50 μm; zoom-in: 15 μm. (D) Comparison of the transduction efficiency (IIba1/eGFP-double-positive/total Iba1$^+$ cell numbers) in OPL$_{microglia}$ (Wilcoxon rank-sum test, $p = 0.452$) and (G) IPL$_{microglia}$ (two-sample t test, $p = 0.354$). (E and H) Comparison of microglial transduction between ROIs for OPL$_{microglia}$ (E, Wilcoxon signed-rank test: TYY, $p = 0.001$; Δ4, $p = 0.005$) or IPL$_{microglia}$ niche (H, Wilcoxon signed-rank test: TYY, $p = 0.092$; Δ4, $p = 0.003$). TYY: 17 retinas, 9 mice. Δ4: 11 retinas, 6 mice. $^*p < 0.05$, $^{ns}p > 0.05$.

based on previously reported "Cre-leakiness" in the *Cx3cr1*^{CreERT2} model.[46] Furthermore, the absence of the Cre recombinase in C57BL6/J animals prevented DIO-mediated inversion and eGFP expression (Figure S8A). We also assessed whether transduced microglia exhibited a different reactivity profile compared to neighboring non-transduced cells. We found no significant differences in OPL$_{microglia}$ between CD68 expression, which increases as a cell enters a more reactive state,[47] or in cell morphology based on Sholl analysis (Figures S8B and S8C).[48] IPL$_{microglia}$ showed small differences, which may be attributed to the small number of transduced cells in the IPL (Figure S8D).

Minimal off-target transgene expression remained with the combination of DIO-AAV and *Cx3cr1*$^{CreERT2/+}$ mice. Within ROI1, only the ONL showed 1–2 off-target cells in 3 out of 13 retinas (Figure 5E). This could be attributed to spontaneous transgene inversion during AAV production.[49] Indeed, we observed similar off-target expression in the ONL of both *Cx3cr1*$^{CreERT2/+}$ mice without tamoxifen and C57BL6/J with tamoxifen (Figure 5D).

Together, the combined approach using scAAV2/6$^{\Delta4}$-CD68-DIO-eGFP and *Cx3cr1*$^{CreERT2/+}$ mice confirms microglial-specific transgene expression with minimal off-target labeling.

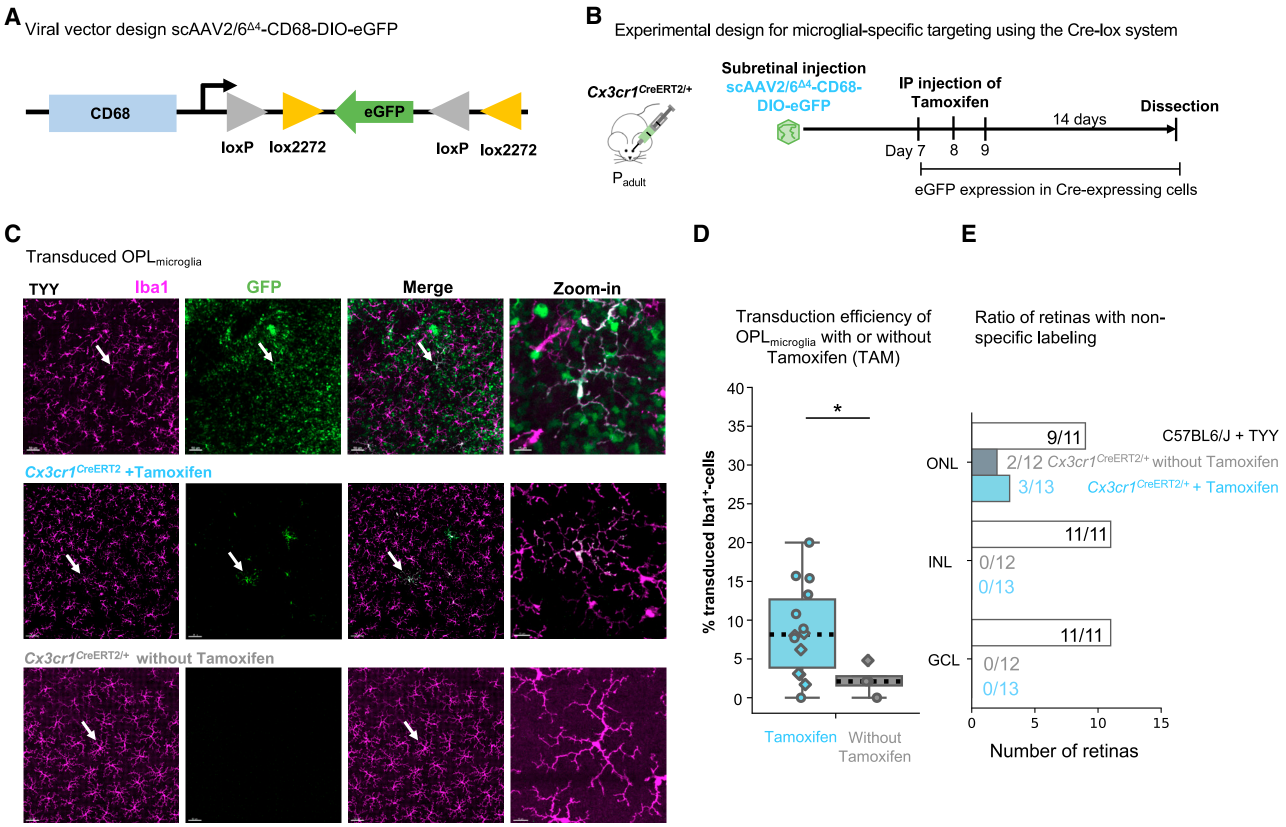

Figure 5. Microglia-specific transgene expression using Cre-dependent scAAV2/6$^{\Delta4}$

(A) Transfer vector design. Two loxP sites flank the inverted eGFP transgene. (B) Experimental timeline. Adult *Cx3cr1*$^{CreERT2/+}$ mice received subretinal injection of scAAV$^{\Delta4}$-CD68-DIO-eGFP (3 $\times$ 10^{12} gc/mL) and tamoxifen injections for 3 consecutive days, 1 week after viral injection. Two weeks later retinas were collected. (C) Retinal whole mounts of transduced OPL$_{microglia}$ of C57BL6/J mice after subretinal injection of scAAV^{TYY}-CD68-DIO-eGFP (Figure 1) and *Cx3cr1*$^{CreERT2/+}$ mice after subretinal injection of scAAV$^{\Delta4}$-CD68-DIO-eGFP with and without receiving tamoxifen treatment. White arrows indicate zoom-in. Scale bar: 50 μm; zoom-in: 15 μm. (D) Comparison of transduction efficiency (Iba1/eGFP-double-positive/total Iba1$^+$ cell numbers) in the OPL with and without tamoxifen treatment (Wilcoxon rank-sum test, $p = 0.038$). (E) Ratio of analyzed retinas showing off-target eGFP expression in the indicated retinal layers. Dataset for comparison. *Cx3cr1*$^{CreERT2/+}$ with tamoxifen: 14 retinas, 9 mice. *Cx3cr1*$^{CreERT2/+}$ without tamoxifen: 4 retinas, 3 mice. *$p < 0.05$. loxP, locus of X-over P1; CreERT2, tamoxifen-inducible Cre recombinase; *Cx3cr1*, CX3C chemokine receptor 1; DIO, double-floxed inverse orientation; IP, intraperitoneal; TYY, scAAV2/6^{TYY}.

Optimization of microglial transduction using Cre-dependent AAV2/6$^{\Delta4}$

Using the retina as a model environment to establish and validate *in vivo* microglia-specific targeting allowed us to further refine technical aspects of our system.

One technical challenge could be cross-recombination when using the Cre-dependent AAV in tandem with a floxed reporter mouse line.[50] To estimate the likelihood of cross-recombination, which can result in loss of the reporter transgene expression, we subretinally injected scAAV2/6$^{\Delta4}$-CD68-DIO-eGFP into *Cx3cr1*$^{CreERT2/+}$/Rosa26$^{Ai9/+}$ tdTomato reporter mice.[51] In this mouse model, microglia express tdTomato upon tamoxifen injection (Figure 6A). Less than 5% of the transduced eGFP$^+$ microglia lacked tdTomato expression in both plexiform layers (Figure 6B), suggesting that cross-recombination occurs very rarely in this reporter line.

Another technical consideration is viral titer, which influences both transduction efficiency and ocular toxicity *in vivo*.[29,52] Thus, we compared how subretinal injection of different viral titers impacted OPL$_{microglia}$ transduction efficiency using the scAAV2/6$^{\Delta4}$-CD68-DIO-eGFP in adult *Cx3cr1*$^{CreERT2/+}$ animals. A viral titer of 3 $\times$ 10^{12} genome copies (gc)/mL resulted in 8% OPL$_{microglia}$ transduction (Figure 6C), while halving the titer to 1.5 $\times$ 10^{12} gc/mL led to 14% microglial transduction. Further reduction of the injection volume to a titer of 9 $\times$ 10^{11} gc/mL slightly enhanced OPL$_{microglia}$ transduction to 17%, suggesting that lower titer might be beneficial for subretinal injections targeting microglia.

One major limitation of self-complementary AAVs is the reduced packaging size, which enhances transgene expression[53] but, on the other hand, halves the AAV packaging size. Thus, we generated a single-stranded AAV (ssAAV) vector and subretinally injected

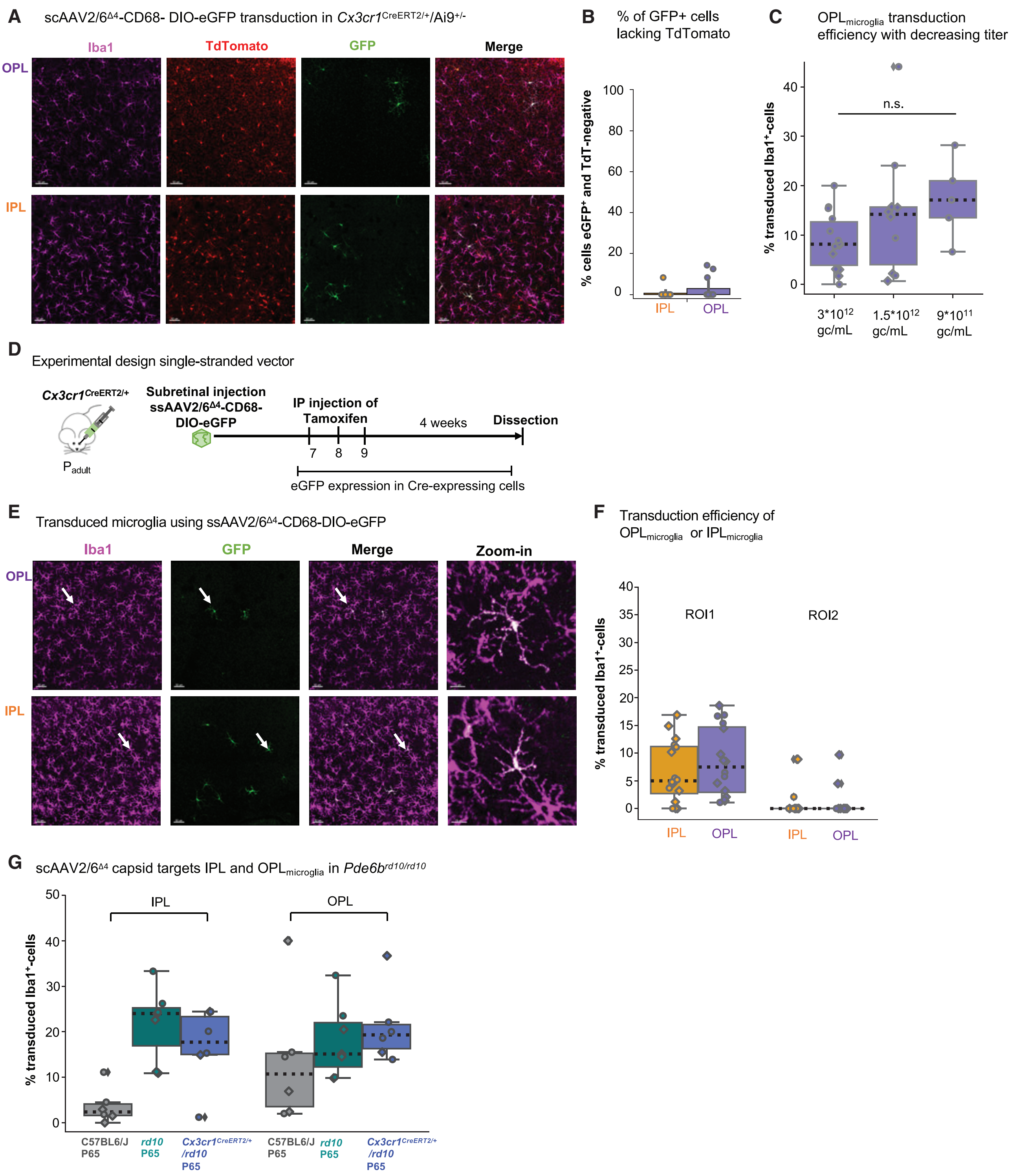

Figure 6. Optimization of microglia targeting using AAV2/6$^{\Delta4}$-CD68-DIO-eGFP

(A) Retinal whole mounts of the OPL$_{microglia}$ and IPL$_{microglia}$ of *Cx3cr1*$^{CreERT2/+}$/Ai9$^{+/-}$ mice after subretinal injection of scAAV$^{\Delta4}$-CD68-DIO-eGFP and tamoxifen treatment. Scale bar: 50 μm. (B) Quantification of co-expression of eGFP and TdTomato. (C) Comparison of OPL$_{microglia}$ transduction efficiency (Iba1/eGFP-double-positive/total Iba1^{+} cell numbers) using different viral titers (Kruskal-Wallis test: p = 0.127). (D) Experimental timeline. *Cx3cr1*$^{CreERT2/+}$ mice received subretinal injection of ssAAV$^{\Delta4}$-CD68-DIO-eGFP and tamoxifen injections for 3 consecutive days 1 week after viral injection. 4 weeks after the first tamoxifen injection the retinas were collected. (E) Retinal whole mounts

(legend continued on next page)

ssAAV2/6$^{\Delta4}$-CD68-DIO-eGFP into the retina. The overall transduction efficiency was consistent with the scAAV2/6$^{\Delta4}$-CD68-DIO-eGFP (Figures 6D–6F), indicating that the self-complementary genome does not contribute to significant differences in transgene expression. The ssAAV can allow a size up to 3 kb for the transgene after exclusion of promoter, ITR, and polyA sequences.

Finally, the retina allows for investigation of niche-selective microglial targeting. Our AAV2/6$^{\Delta4}$ capsid showed preference for OPL$_{microglia}$, while IPL$_{microglia}$ transduction remained low (Figure 4G; Figures S8B and S8C). Since we found a significant increase in IPL$_{microglia}$ transduction in the P65 *Pde6b$^{rd10/rd10}$* condition, we questioned whether a potential niche-selective expression of the scAAV2/6$^{\Delta4}$ capsid remained (Figure 3C). Thus, we crossed the *Cx3cr1*$^{CreERT2/+}$ onto the *Pde6b$^{rd10/rd10}$* background, subretinally injected scAAV2/6$^{\Delta4}$-CD68-DIO-eGFP, and compared the results to scAAV2/6^{TYY}-CD68-eGFP from Figure 3C. ScAAV2/6$^{\Delta4}$ transduced IPL$_{microglia}$ at a similar level to scAAV2/6^{TYY} (Figure 6G), suggesting that the AAV2/6$^{\Delta4}$ capsid is capable of transducing both retinal microglial niches.

Taken together, we demonstrated the efficacy of the Cre-dependent AAV2/6$^{\Delta4}$ and validated the viral tool for microglial transduction in the retina.

DISCUSSION

In this study, we show that retinal microglia can be successfully targeted using AAV and are influenced by delivery route. We identified that during photoreceptor degeneration, microglial transduction improved and used this result to inform generation of a modified AAV2/6$^{\Delta4}$ that enhanced OPL$_{microglia}$ in adult animals. Finally, we optimized several parameters to improve AAV2/6$^{\Delta4}$ for future microglial transduction studies.

Transduction of microglia in degenerative conditions

In two retinal degenerative conditions, we were able to use known environmental changes to dissect parameters that may affect microglial transduction efficiency. ONC disrupts the inner limiting membrane and reduces the nerve fiber layer thickness.[32] We expected that this would improve AAV2/6 access to reach the retinal layers and therefore microglial transduction; however, this was not the case. One explanation could be the difference in AAV2 and AAV6 capsid tropism and heparin-binding affinity. AAV2 capsid has a high affinity for heparin and accumulates at the inner limiting membrane, which requires HSPG binding to pass through.[38,54] In contrast, AAV6 uses both HSPG and sialic acid for cellular attachment and entry and displays a weaker heparin-binding affinity than AAV2 capsid.[41,55] This suggests that AAV6 is inferior to AAV2 at spreading evenly throughout the retina after intravitreal delivery,[56,57] and therefore it may not gain an advantage to targeting cells after ONC.

In contrast to ONC, subretinal injection in the *Pde6b$^{rd10/rd10}$* resulted in a robust microglial transduction (Figure 3). The virus is trapped in the subretinal space, where the weaker heparin-binding affinity of AAV2/6[41,55] may assist its propagation through the subretinal space before cell attachment. Furthermore, extracellular matrix remodeling, accompanied by the reduction of the densely packed ONL, could affect viral cell attachment and transduction in the subretinal space.[58]

The surprising finding was the significant increase in IPL$_{microglia}$ transduction in the P65 *Pde6b$^{rd10/rd10}$* environment (Figure 3). The reason for this could be two-fold: either the extracellular matrix has already been restructured in the INL, allowing easier access of AAV to the IPL$_{microglia}$, and/or a multifaceted glial response has adapted to the changing degenerative environment. Microglia are known to take on new and distinct transcriptional states in degenerative conditions.[22,59] The increase in IPL$_{microglia}$ transduction from P27 to P65 *Pde6b$^{rd10/rd10}$* could suggest that microglia become more susceptible to transduction throughout disease progression, which is an interesting observation for follow-up studies.

Nonspecific labeling

Off-target transgene expression is an ongoing challenge in viral gene delivery and can only be circumvented by optimizing viral tropism and cell-type-specific promoters. Both aspects are ill-defined for microglia. Besides AAVs, lentiviral vectors have been used to target microglia, but they prevent off-target transgene expression upon employing a microRNA-9 sponge.[60] This strategy is suboptimal, because microRNA-9 has known effects on neurogenesis and synaptic plasticity;[61,62] thus, sequestering microRNA-9 in off-target cells could result in unknown effects that confound experimental results. Here, we have focused on AAV2/6 due to its suggested specificity in targeting microglia *in vivo*.[14] However, we found that scAAV2/6^{YTT}-CD68-eGFP resulted in strong off-target cell type labeling in the retina. Implementing a tamoxifen-inducible system combining *Cx3cr1*^{CreERT2} animals with scAAV2/6$^{\Delta4}$-DIO-CD68-eGFP led to microglia-specific labeling in the GCL and INL (Figure 5I). Only in the ONL, we found non-specific labeling of 1–5 cells per ROI in only a few retinas analyzed. We suspect that this is due to spontaneous inversion of DIO transgenes,[49] which we substantiated in our control experiments (Figure 5D). To eradicate off-target expression, incorporation of mutant recombinase recognition sites can prevent the spontaneous inversion.[49]

Nevertheless, the microglial community is in need of microglia-specific promoters. The rapid environmental adaptation of microglia is reflected by transcriptional changes, which makes it challenging to identify reliable promoters that encompass all potential microglial conditions. Promoters such as *CD68* and *Cx3cr1* also label blood-derived or brain-barrier-associated macrophages.[63,64] Proposed new

of OPL$_{microglia}$ and IPL$_{microglia}$ of *Cx3cr1*$^{CreERT2/+}$ mice after subretinal injection of ssAAV$^{\Delta4}$-CD68-DIO-eGFP and tamoxifen treatment. White arrows indicate zoom-in. Scale bar: 50 μm; zoom-in: 15 μm. (F) Comparison of microglial transduction efficiency between ROIs for both OPL and IPL niche after subretinal delivery of ssAAV2/6$^{\Delta4}$-CD68-eGFP. (G) Comparison of transduction efficiency in the OPL$_{microglia}$ and IPL$_{microglia}$ of C57BL6/J, *Pde6b$^{rd10/rd10}$* (rd10) (Figure 3 dataset) and *Cx3cr1*$^{CreERT2/+}$/*Pde6b$^{rd10/10}$* mice after subretinal virus delivery. $^{ns}p > 0.05$. ssAAV, single-stranded adeno-associated virus.

markers from RNA sequencing studies such as transmembrane protein 119 (TMEM119) have been recently challenged on their specificity (e.g., in the retina).[65] On the other hand, the retina provides a unique opportunity to explore novel viral transduction strategies and validating new promoters in future studies.[16]

Variability of microglial transduction efficiency

Across CNS regions and viral types, microglial transduction remains at low efficiencies and variable within conditions.[5] We observed variation within experimental groups throughout our work, which was independent from injection method, capsid variants, Cre-dependent genomes, or animal sex. Interestingly, in our cross-validation fluorescence-activated cell sorting (FACS) experiments, we also saw significant variability among transduction efficiencies of microglia, even though the overall percentage of eGFP$^+$ cells (microglia and non-microglia) did not vary (Figures S1G and S1J). These variations appear in other studies, yet without further discussion of the source of the variation.[66,67]

Transcriptomic studies have made it clear that the microglial population is highly heterogeneous.[59,68] On the one hand, microglia maintain a common gene signature, which overlaps with other immune cells; yet on the other hand, microglia adapt to their local CNS niche, which might manifest into more distinctly heterogeneous populations after damage.[22,69] Since we know microglia respond to both the injection-induced insult and to the AAV particles themselves,[29] we may create a new microglial niches, which contribute to an overall increase in heterogeneity across the microglial population. One way to mitigate these effects could be to deliver the virus alongside compounds known to reduce microglia reactivity, for example, translocator protein (TSPO)[70] or minocycline;[71] however, the effects on the experimental condition must also be considered.

Conclusion

Our work highlights the feasibility of microglial transduction in the retina with a modified AAV2/6. Using retinal degeneration models to assess the effect of altered environments on microglial transduction, we found enhanced microglia targeting in a photoreceptor degenerative model. We applied this finding to generate a modified AAV2/6 that increases $OPL_{microglia}$ transduction in healthy adult animals. Finally, we validated and optimized a Cre-dependent AAV strategy for specific microglial targeting *in vivo* that provides the foundation for future studies.

MATERIALS AND METHODS

Cloning

The self-complementary transfer vector (pAAV2-CD68-MVMi-DIO-eGFP) was generated using the pAAV2-CD68-MVMi-hGFP plasmid kindly provided by Rosario et al.[14] and an RV-CAG-DIO-eGFP plasmid, purchased from Addgene (87662). Both plasmids were digested with *Sac*I and *Pst*I (New England Biolabs) to obtain fragments containing the vector backbone *CD68* promoter and the DIO-eGFP insert, which were ligated to yield the final product, pAAV2-CD68-MVMi-DIO-eGFP. The single-stranded (ss) pAAV2-CD68-MVMi-DIO-eGFP plasmid was generated by PCR amplification of the insert containing *CD68* promoter and loxP sites flanking the eGFP using primers CD68-DIO-eGFP For and CD68-DIO-eGFP Rev (Table 1) from the pAAV2-CD68-MVMi-DIO-eGFP plasmid. The resulting product, along with ssAAV backbone (pAAV-ProA3(SynP137)-ChR2d-eGFP-WPRE), kindly provided by Botond Roska, was digested with *Mlu*I and *Rsr*II (New England Biolabs, R3198S, R0501S). The ligated product was transformed and purified, then confirmed by Sanger sequencing.

Table 1. List of primers.

Primer	Sequence (5′–3′)
DIO-eGFP For	ACAGCGCTGCAGATAACTTCGTATAGGA TACTTTATACGAAGTTATGCAGA
DIO-eGFP Rev	ACAGCGGCTAGCACAAATTTTGTAATCC AGAGGTTGATTGGTTTAAAC
CD68-DIO-eGFP For	ATCACGCGTCGTGGATCTGAATTCAATTCA
CD68-DIO-eGFP Rev	ATTCGGTCCGCATGCCTGCTATTG
AAV6 CAP R576Q For	GGCCACCGAACAATTTGGGACTGTG
AAV6 CAP R576Q Rev	ACGGGGTTAGTGGCTTTG
AAV6 CAP K531E For	CAAAGACGACGAAGACAAGTTCTTTC
AAV6 CAP K531E Rev	TGTGAGGCCATAGCAGTG
AAV6 CAP K459S For	GCCCAAAACAGCGACTTGCTGTTTAG
AAV6 CAP K459S Rev	ACTTCCGGACTGATTCTG
AAV6 CAP K493S For	TCTAAAGTAAGCACAGACAACAACAA CAGCAACTTTACCTGGAC
AAV6 CAP K493S Rev	AACGCGCTGCTGCCGGTA

AAV, adeno-associated virus; CAP, capsid; CD68, cluster of differentiation 68; DIO, double-floxed inverse orientation; eGFP, enhanced green fluorescent protein; FOR, forward primer; REV, reverse primer.

Site-directed mutagenesis

Single-nucleotide substitutions were performed using the Q5 Site-Directed Mutagenesis Kit from New England Biolabs (E0552S). Exponential amplification of the viral plasmid capsid (pACGr2c6-T492V-Y705F-Y731F) provided by Rosario et al.[14] was performed according to the manufacturer's instructions. Each mutagenesis PCR reaction was performed subsequently on the resulting plasmid from the previous confirmed mutagenesis reaction using the primer pairs listed in Table 1. Mutant vectors were transformed in chemically competent DH5α cells (Invitrogen) and DNA isolated using Monarch mini-prep DNA kits. Mutations were confirmed by sequencing, and alignment was performed with SnapGene 4.1.9.

AAV production

Transfection of HEK293T cells

Human embryonic kidney (HEK) 293T cells were purchased from the American Type Culture Collection (ATCC) and maintained at 37°C in 5% (v/v) CO_2 in complete medium (DMEM high-glucose GlutaMAX supplement pyruvate [Thermo Fisher Scientific, 31966047], 10% [v/v] fetal bovine serum [FBS] [Gibco Fetal Bovine Serum, qualified, E.U.-approved, South America origin, Thermo Fisher Scientific 10270106], 1% [v/v] penicillin/streptomycin [stock: 10,000 U/mL,

Table 2. List of AAVs

Virus name	Transfer vector	Capsid	Titer (gc/mL)
scAAV2/6^{TYY}-CD68-eGFP	pAAV2-CD68-MVMi-eGFP	pACG r2c6-T492V+Y705F+Y731F	1.37×10^{11} 1.06×10^{12}<br>1.94×10^{11}
scAAV2/6^{K531E}-CD68-eGFP	pAAV2-CD68-MVMi-eGFP	pACG r2c6-T492V+Y705F+Y731F+K531E	5.59×10^{12}
scAAV2/6$^{\Delta 4}$-CD68-eGFP	pAAV2-CD68-MVMi-eGFP	pACG r2c6-T492V+Y705F+Y731F+K531E+ K459S+R576Q+K493S	1.17×10^{13}
scAAV2/6$^{\Delta 4}$-CD68-DIO-eGFP	pAAV2-CD68-MVMi-DIO-eGFP	pACG r2c6-T492V+Y705F+Y731F+K531E+ K459S+R576Q+K493S	3×10^{12}
ssAAV2/6$^{\Delta 4}$-CD68-DIO-eGFP	pAAV2-CD68-MVMi-DIO-eGFP	pACG r2c6-T492V+Y705F+Y731F+K531E+ K459S+R576Q+K493S	3.13×10^{12}

Each AAV was produced with the corresponding transfer vector and capsid. Resulting titer(s) are notated in genome copies per milliliter (gc/mL). scAAV, self-complementary adeno-associated virus; CD68, cluster of differentiation 68; DIO, double-floxed inverse orientation; eGFP, enhanced green fluorescence protein; MvMi, minute virus of mice intron.

Thermo Fisher Scientific, 15140122], 1% [v/v] non-essential amino acids [stock: 100×, Sigma-Aldrich, M7145-100ML]). Ten T150 flasks were seeded to reach 80% confluency for the day of transfection. High yield of plasmid DNA was obtained using the NucleoBond Xtra Maxi Plus EF (Macherey-Nagel, 740426.50). 70 μg AAV packaging plasmid, 70 μg AAV vector plasmid, and 200 μg helper plasmid were added to 50 mL DMEM without serum, followed by 1,360 μL PEI (polyethylenimine, Polysciences, 24765-2). 5 mL of DNA-transfection mixture was added to each T150 flask after 15 min incubation.

AAV isolation

60 h after transfection, the cells were dislodged, pelleted, and stored at −80°C. For AAV isolation, cells were resuspended in lysis buffer (150 mM NaCl, 20 mM Tris [pH 8.0], sterile filtered) and subject to three rounds of freeze/thaw cycles between dry ice/ethanol bath and 37°C water bath. $MgCl_2$ was added (final concentration 1 mM), followed by Turbonuclease (final concentration 250 U/mL, BPS Bioscience, BPS 50310) to remove contaminating plasmid and genomic DNA. The cell suspension was spun down at 4,000 rpm at 4°C for 20 min, at which point the viral fraction was in the supernatant.

The virus was purified by discontinuous iodixanol gradient ultracentrifugation.[72] Optiseal tubes (Beckman Coulter, 361625) were filled with a density gradient of 60%, 40%, 25%, and 17% iodixanol solutions (Optiprep Iodixanol, Progen Biotechnik, 1114542).

The viral lysate supernatant was loaded on the top layer and the tubes were centrifuged at 242,000 × *g* at 16°C for 90 min in a Beckman Optima XPN-100 ultracentrifuge, using a 70Ti rotor. The AAV particles were harvested from the intersection of 60% and 40% gradients and purified and concentrated using Amicon filters (Millipore Amicon 100K, Merck, UFC910008). 20 μL aliquots were stored at −80°C, and a 5 μL aliquot was reserved for titration by qPCR. The DNase- (New England Biolabs, M0303S) treated virus aliquot was serially diluted (1:10 to 1:100,000) and run alongside a linearized standard template DNA (1×10^{10}–1×10^{3} gc). The Luna universal qPCR Master mix (New England Biolabs, M3003L) was prepared according to the manufacturer's instructions with a reaction volume of 10 μL and run on a BioRad C1000 cycler using the following primers to amplify a 100 bp product within the bovine growth hormone polyadenylation signal (BGHpA): forward: 5′-CCAGCCATCTGTTGT TTGC-3′; reverse: 5′-ACAATGCGATGCAATTTCC-3′. The viral genome copy number per milliliter (gc/mL) was calculated as previously described by Pfaffl.[73] All AAVs produced for this study are listed in Table 2. Viruses were diluted when required to use consistent titers for experimental and control groups for accurate comparisons.

Primary mixed glia culture

Mixed glia cultures were prepared as detailed by Bronstein et al.[74] Briefly, cortices were dissected from 3–5 murine pups aged P0–P2 in ice-cold Hank's buffered saline, then digested in 0.05% Trypsin + EDTA (1×) for 15 min at 37°C. The digestion was neutralized by adding (v/v) serum-containing medium (DMEM, 10% FBS, 1% penicillin/streptomycin, 1% non-essential amino acids), and the cells were pelleted at 500 × *g* for 5 min. After one wash, the cell pellet was resuspended in 15 mL of medium and passed through a 40 μm cell strainer. This cell suspension was plated directly onto an ibidi 8-well chamber slide (200 μL/well). The culture medium was replaced after the third day, and at day 10 the mixed glia culture was mature for further experiments. For viral transduction, 1×10^{8} viral genome copies were added per well of an 8-well ibidi chamber slide (growth area, 1 cm^2).

Animals

As indicated throughout the study, mice of both sexes and ages (4–17 weeks) were used. Founder animals were purchased from The Jackson Laboratory for the following strains: C57BL6/J (000664), *Pde6b*$^{rd10/rd10}$ (004297), *Cx3cr1*^{CreERT2} (020940), and *Cx3cr1*^{GFP} (005582).[8] All mice were backcrossed to the C57BL6/J background for at least 10 generations. Animals were housed and maintained in the IST Austria Preclinical Facility, with 12 h light-dark cycle and food and water provided *ad libitum*. All animal procedures were approved by the Bundesministerium für Wissenschaft, Forschung und Wirtschaft (bmwfw) Tierversuchsgesetz 2012, BGBI. I Nr. 114/2012 (TVG 2012) under the number GZ BMWFW-66.018/

Table 3. Antibody dilutions

Antibody	Dilution	Company
Anti-GFP chicken	1:500	Thermo Fisher Scientific (A10262)
Anti-Iba1 goat	1:250	Abcam (ab 5076)
Anti-Iba1 rabbit	1:500	GeneTex (GTX100042)
Anti-RBPMS rabbit	1:200	Abcam (194213)
Anti-Cone arrestin rabbit	1:500	Sigma-Aldrich (AB15282)
Anti-GFAP rabbit	1:300	Dako (Z 0334)
Alexa Fluor 488 donkey anti-chicken IgY	1:500	Sigma-Aldrich (SAB 4600031-250 μL)
Alexa Fluor 568 donkey anti-rabbit IgG (H+L)	1:2,000	Thermo Fisher Scientific (A10042)
Alexa Fluor 647 donkey anti-goat IgG (H+L)	1:2,000	Thermo Fisher Scientific (A21447)
Alexa Fluor 647 goat anti-rabbit IgG (H+L)	1:2,000	Thermo Fisher Scientific (A31573)
Alexa Fluor 488 goat anti-chicken IgG (H+L)	1:2,000	Thermo Fisher Scientific (A11041)
Anti-CD11b mouse eFluor 660	6 μg/mL	Thermo Fisher Scientific (50-0112-82)
Anti-CD45 mouse eFluor 450	15 μg/mL	Thermo Fisher Scientific (48-0451-82)
Anti-CD16/CD32 monoclonal Ab	1 μg/mL	Thermo Fisher Scientific (14-0161-85)

CD11b/ITGAM, integrin alpha M; CD16/CD32, Fc fragment of IgG; CD45/PTPRC, protein tyrosine phosphatase, receptor type, C; Iba1, ionized calcium-binding adaptor molecule 1; GFAP, glial fibrillary acidic protein; GFP, green fluorescent protein.

005-WF/V3b/2016 and by IST Austria Ethics Officer. For tamoxifen administration, *Cx3cr1*$^{CreERT2/+}$ and C57BL6/J mice received intraperitoneal (i.p.) injections of tamoxifen (Sigma Aldrich, T5648-5G) dissolved in corn oil (Sigma Aldrich, C8267-500ML, 150 mg/kg body weight, 20 mg/mL stock solution) at the age of 4–6 weeks once per day for 3 consecutive days.

Anesthesia and surgical preparation

Mice were anesthetized with 5% (v/v) isoflurane (Zoetis) supplemented with oxygen at a flow rate of 0.6 L/min. The anesthetized mice were transferred to a heating pad placed under a Leica dissection microscope housed in a biosafety cabinet and subsequently maintained at 2.5% (v/v) isoflurane supplemented with oxygen via a nose cone during the procedure. Proparacaine (0.5% HCl) eye drops (Ursapharm Arzneimittel) were applied to numb the eyes, and subcutaneous injection of 100 μL Metacam (Meloxacam, Boehringer Ingelheim) per 25 g mouse (5 mg/kg) alleviated pain.

Ocular injections

A jeweler's forceps was used to grasp the conjunctiva, then the sclera was carefully punctured with the bevel of a 27G (Henry Schein Medical) needle just below the limbus. A Nanofil syringe equipped with a 35G blunt-ended needle (World Precision Instruments) was inserted via the pre-punctured hole. For trans-scleral subretinal injections, the syringe needle was inserted with care to avoid the lens and continued until resistance could be detected indicating passage through the retinal tissue. A slight retraction of the needle allows the syringe content to be released into the subretinal space of the inferotemporal or superotemporal quadrant for the right or left eyes, respectively. For intravitreal injections, the needle was inserted 1–2 mm into the eye. Once inserted in either method, 1 μL virus was slowly released over 30 s into the subretinal space or into the vitreous body, and the syringe remained in position for an additional 45 s. Triple-antibiotic ointment was applied to the eye after the procedure. Injections were carried out on the same day for control and experimental groups (e.g., ONC versus non-crushed naive controls). Comparison groups are indicated in figure legends.

Optic nerve crush (ONC)

The lateral canthus of the left eye was pinched for 10 s using a hemostat, then a lateral canthotomy was performed to allow visualization of the posterior pole. A jeweler's forceps was used to firmly securely the eye at the limbus of the conjunctiva. A micro-dissection scissors was used to cut the conjunctiva in both the superior and inferior direction. To expose the optic nerve, a window was created by carefully dissecting the surrounding muscle and fascia. The optic nerve was then pinched 1 mm from the posterior pole for 4 s using a curved N5 self-closing forceps (Dumont). Triple-antibiotic ointment was applied to the eye to prevent infection.

Retina preparation and immunostaining

Transduction efficiency was assessed at 2 weeks post injection, which should be sufficient to reach high expression with the self-complementary AAV.[75] Following cervical dislocation and decapitation, eyes were enucleated with curved forceps. Retinas were rapidly dissected in 1× phosphate-buffered saline (PBS) and transferred to 4% (w/v) paraformaldehyde (Sigma-Aldrich, P6148-1KG) for 30 min fixation. After 3× wash in 1× PBS, retinas were placed overnight at 4°C in 30% (w/v) sucrose (Sigma-Aldrich, 84097-1KG)/PBS. After three freeze-thaw cycles on dry ice, retinas were washed three times with 1× PBS, and blocked for 1 h at room temperature (RT) in blocking solution (1% [w/v] bovine serum albumin [Sigma A9418], 5% [v/v] Triton X-100 [Sigma T8787], 0.5% [w/v] sodium azide [VWR 786-299], and 10% [v/v] serum [either goat, Millipore S26, or donkey, Millipore S30]).

For immunostaining, primary antibodies were diluted in antibody solution containing 1% (w/v) bovine serum albumin, 5% (v/v) Triton X-100, 0.5% (v/v) sodium azide, 3% (v/v) goat or donkey serum for at least 3 days at 4°C on a shaker. The dilution factors of the antibodies are shown in Table 3. After washing, the retinas were incubated light-protected with secondary antibodies (Table 3) diluted in antibody solution for 2 h at room temperature on a shaker. The retinas were washed three times with 1× PBS for 30 min. The nuclei were labeled with Hoechst 33342 (1:5,000, Thermo Fisher Scientific, H3570) in 1× PBS for 10 min at room temperature and washed again three times with 1× PBS for 30 min. The retina was whole mounted on a glass cover slide with the ganglion cell layer facing up, and antifade solution containing 10% (v/v) Mowiol (Sigma, 81381), 26% (v/v) glycerol

(Sigma, G7757), 0.2 M Tris buffer (pH 8, 2.5%) (w/v) Dabco (Sigma, D27802) was added and a coverslip applied (#1.5 VWR, 631-0147).

Exclusion criteria

We excluded retinas from the analysis when (1) the injected eye exhibited cataract formation due to damage of the lens during injection, (2) retinal hemorrhage during the injection procedure was visible after dissection, or (3) the immunostaining showed amoeboid-shaped Iba1$^+$ cells located outside of the plexiform layers either in the nerve fiber layer or the outer nuclear layer indicating infiltrating macrophage due to injection damage. The overall exclusion incidence was between 5% and 10%.

Retina dissociation and flow cytometry

Animals were briefly anesthetized with isoflurane (Zoetis, 6089373), decapitated, and the retina was immediately explanted and dissected on ice in Hanks' balanced salt solution (HBSS, Thermo Fisher 14175053). After dissection, retinas were enzymatically dissociated in the pre-warmed digestion solution; 1:8:1 cysteine/EDTA solution (2.5 mM cysteine, 0.5 mM EDTA [ethylenediaminetetraacetic acid] in HBSS), 10 mM HEPES (4-(2-hydroxyethyl)-1-piperazineethanesulfonic acid) in HBSS, and papain (10 mg/mL Roche 10108014001) for 10 min at 37°C. Samples were then centrifuged for 2.5 min at 1,600 rpm. The supernatant was discarded, and cells were washed twice with 1 mM EDTA in HBSS + 2% (v/v) FBS. The digested tissue was then mechanically dissociated through a pulled glass pipette.

Non-antigen-specific binding was blocked by incubating each sample with CD16/CD32 monoclonal antibody (Table 3) in 1 mM EDTA in HBSS + 2% (v/v) FBS solution on ice for 10 min. After washing once with 1 mM EDTA in HBSS + 2% (v/v) FBS, cells were incubated on ice for 30 min with fluorophore-conjugated antibodies against CD11b and CD45 (see Table 3). After the incubation, samples were washed once with ice-cold 1 mM EDTA in HBSS + 2% (v/v) FBS, filtered through a 70 μm strainer, and transferred to the flow cytometer (Sony SH800SFP).

Non-stained and single-stained samples were used to set auto-fluorescence thresholds and to compensate dyes, which was necessary due to overlapping emission wavelengths. Compensation was carried out with the Compensation Wizard from the SH800 flow cytometer software and then applied to each sample recording. Populations of interest were gated, and at least 100,000 events were recorded for each sample. All data analysis was performed using the FlowJo software (v.10.7.2; FlowJo, Ashland, OR, USA).

Confocal microscopy and image analysis

Flat-mounted retinas were imaged with an Axio Imager Z2 Zeiss LSM800 upright confocal microscope using a Nikon Plan-Apochromat 20× magnification air objective (NA 0.8). A 2 × 2 tile scan image with Nyquist z-steps was acquired in two ROIs of retina, each measuring 608 × 608 μm when stitched. All images were acquired using the same settings. Stitched tile scans were analyzed in Imaris v.9.3 using the spots function to facilitate cell counting. Transduction efficiency of microglia was analyzed in the outer and the inner plexiform layers of the retina. Transduction efficiency was calculated by dividing the number of transduced Iba1$^+$ cells (Iba1/eGFP-double-positive cells) by the total number of Iba1$^+$ cells within an ROI. Sholl analysis was determined by the number of filament Sholl intersections exported from a 3-dimensional microglial trace using the Filament tracing plug-in in Imaris v.9.3. CD68 volume was determined using the 3-dimensional surfaces function in Imaris v.9.3 based on CD68 immunostaining present within microglia. The total volume of CD68 was reported as a percentage of total microglial volume within an ROI.

Statistical analysis

All statistics were performed using the statistical functions in SciPy library (v.1.6.2) in python as indicated in the figure legends. Retinas were excluded from analysis if a cataract was present at the time of retinal dissection or if high macrophage infiltration in the tissue was observed, indicating significant tissue damage from the injection. Error bars represent the standard error of the mean.

SUPPLEMENTAL INFORMATION

Supplemental information can be found online at https://doi.org/10.1016/j.omtm.2021.09.006.

ACKNOWLEDGMENTS

This project has received funding from the European Research Council (ERC) under the European Union's Horizon 2020 research and innovation programme (grant agreement no. 715571). The research was supported by the Scientific Service Units (SSU) of IST Austria through resources provided by the Bioimaging Facility, the Life Science Facility, and the Pre-Clinical Facility, namely Sonja Haslinger and Michael Schunn for their animal colony management and support. We would also like to thank Chakrabarty Lab for sharing the plasmids for AAV2/6 production. Finally, we would like to thank the Siegert team members for discussion about the manuscript.

AUTHOR CONTRIBUTIONS

M.E.M. and S.S. conceived and developed the experimental design and wrote the manuscript. M.E.M., G.M.W., G.C., and R.C.-P. performed experiments. M.E.M. and G.M.W. analyzed data, performed statistical analysis, and created figures. All authors read and approved the final manuscript.

DECLARATION OF INTERESTS

The authors declare no competing interests.

REFERENCES

1. Li, C., and Samulski, R.J. (2020). Engineering adeno-associated virus vectors for gene therapy. Nat. Rev. Genet. *21*, 255–272.
2. Chan, K.Y., Jang, M.J., Yoo, B.B., Greenbaum, A., Ravi, N., Wu, W.L., Sánchez-Guardado, L., Lois, C., Mazmanian, S.K., Deverman, B.E., and Gradinaru, V. (2017). Engineered AAVs for efficient noninvasive gene delivery to the central and peripheral nervous systems. Nat. Neurosci. *20*, 1172–1179.
3. Gong, Y., Mu, D., Prabhakar, S., Moser, A., Musolino, P., Ren, J., Breakefield, X.O., Maguire, C.A., and Eichler, F.S. (2015). Adenoassociated virus serotype 9-mediated gene therapy for x-linked adrenoleukodystrophy. Mol. Ther. *23*, 824–834.

4. Cucchiarini, M., Ren, X.L., Perides, G., and Terwilliger, E.F. (2003). Selective gene expression in brain microglia mediated via adeno-associated virus type 2 and type 5 vectors. Gene Ther. *10*, 657–667.

5. Maes, M.E., Colombo, G., Schulz, R., and Siegert, S. (2019). Targeting microglia with lentivirus and AAV: Recent advances and remaining challenges. Neurosci. Lett. *707*, 134310.

6. Siegert, S., Cabuy, E., Scherf, B.G., Kohler, H., Panda, S., Le, Y.Z., Fehling, H.J., Gaidatzis, D., Stadler, M.B., and Roska, B. (2012). Transcriptional code and disease map for adult retinal cell types. Nat. Neurosci. *15*, 487–495, S1–S2.

7. Badimon, A., Strasburger, H.J., Ayata, P., Chen, X., Nair, A., Ikegami, A., Hwang, P., Chan, A.T., Graves, S.M., Uweru, J.O., et al. (2020). Negative feedback control of neuronal activity by microglia. Nature *586*, 417–423.

8. Yona, S., Kim, K.-W., Wolf, Y., Mildner, A., Varol, D., Breker, M., Strauss-Ayali, D., Viukov, S., Guilliams, M., Misharin, A., et al. (2013). Fate mapping reveals origins and dynamics of monocytes and tissue macrophages under homeostasis. Immunity *38*, 79–91.

9. Parkhurst, C.N., Yang, G., Ninan, I., Savas, J.N., Yates, J.R., 3rd, Lafaille, J.J., Hempstead, B.L., Littman, D.R., and Gan, W.B. (2013). Microglia promote learning-dependent synapse formation through brain-derived neurotrophic factor. Cell *155*, 1596–1609.

10. Elmore, M.R.P., Najafi, A.R., Koike, M.A., Dagher, N.N., Spangenberg, E.E., Rice, R.A., Kitazawa, M., Matusow, B., Nguyen, H., West, B.L., and Green, K.N. (2014). Colony-stimulating factor 1 receptor signaling is necessary for microglia viability, unmasking a microglia progenitor cell in the adult brain. Neuron *82*, 380–397.

11. Haynes, S.E., Hollopeter, G., Yang, G., Kurpius, D., Dailey, M.E., Gan, W.-B., and Julius, D. (2006). The P2Y12 receptor regulates microglial activation by extracellular nucleotides. Nat. Neurosci. *9*, 1512–1519.

12. Nectow, A.R., and Nestler, E.J. (2020). Viral tools for neuroscience. Nat. Rev. Neurosci. *21*, 669–681.

13. O'Carroll, S.J., Cook, W.H., and Young, D. (2021). AAV Targeting of Glial Cell Types in the Central and Peripheral Nervous System and Relevance to Human Gene Therapy. Front. Mol. Neurosci. *13*, 618020.

14. Rosario, A.M., Cruz, P.E., Ceballos-Diaz, C., Strickland, M.R., Siemienski, Z., Pardo, M., Schob, K.-L., Li, A., Aslanidi, G.V., Srivastava, A., et al. (2016). Microglia-specific targeting by novel capsid-modified AAV6 vectors. Mol. Ther. Methods Clin. Dev. *3*, 16026.

15. Su, W., Kang, J., Sopher, B., Gillespie, J., Aloi, M.S., Odom, G.L., Hopkins, S., Case, A., Wang, D.B., Chamberlain, J.S., and Garden, G.A. (2016). Recombinant adeno-associated viral (rAAV) vectors mediate efficient gene transduction in cultured neonatal and adult microglia. J. Neurochem. *136* (*Suppl 1*), 49–62.

16. Jüttner, J., Szabo, A., Gross-Scherf, B., Morikawa, R.K., Rompani, S.B., Hantz, P., Szikra, T., Esposti, F., Cowan, C.S., Bharioke, A., et al. (2019). Targeting neuronal and glial cell types with synthetic promoter AAVs in mice, non-human primates and humans. Nat. Neurosci. *22*, 1345–1356.

17. Pandya, J., Ortiz, L., Ling, C., Rivers, A.E., and Aslanidi, G. (2014). Rationally designed capsid and transgene cassette of AAV6 vectors for dendritic cell-based cancer immunotherapy. Immunol. Cell Biol. *92*, 116–123.

18. Hammond, T.R., Dufort, C., Dissing-Olesen, L., Giera, S., Young, A., Wysoker, A., Walker, A.J., Gergits, F., Segel, M., Nemesh, J., et al. (2019). Single-Cell RNA Sequencing of Microglia throughout the Mouse Lifespan and in the Injured Brain Reveals Complex Cell-State Changes. Immunity *50*, 253–271.e6..

19. Gosselin, D., Skola, D., Coufal, N.G., Holtman, I.R., Schlachetzki, J.C.M., Sajti, E., Jaeger, B.N., O'Connor, C., Fitzpatrick, C., Pasillas, M.P., et al. (2017). An environment-dependent transcriptional network specifies human microglia identity. Science *356*, 1248–1259.

20. Butovsky, O., Jedrychowski, M.P., Moore, C.S., Cialic, R., Lanser, A.J., Gabriely, G., Koeglsperger, T., Dake, B., Wu, P.M., Doykan, C.E., et al. (2014). Identification of a unique TGF-β-dependent molecular and functional signature in microglia. Nat. Neurosci. *17*, 131–143.

21. Santos, A.M., Calvente, R., Tassi, M., Carrasco, M.C., Martín-Oliva, D., Marín-Teva, J.L., Navascués, J., and Cuadros, M.A. (2008). Embryonic and postnatal development of microglial cells in the mouse retina. J. Comp. Neurol. *506*, 224–239.

22. O'Koren, E.G., Yu, C., Klingeborn, M., Wong, A.Y.W., Prigge, C.L., Mathew, R., Kalnitsky, J., Msallam, R.A., Silvin, A., Kay, J.N., et al. (2019). Microglial Function Is Distinct in Different Anatomical Locations during Retinal Homeostasis and Degeneration. Immunity *50*, 723–737.e7.

23. Zhang, Y., Chen, K., Sloan, S.A., Bennett, M.L., Scholze, A.R., O'Keeffe, S., Phatnani, H.P., Guarnieri, P., Caneda, C., Ruderisch, N., et al. (2014). An RNA-sequencing transcriptome and splicing database of glia, neurons, and vascular cells of the cerebral cortex. J. Neurosci. *34*, 11929–11947.

24. Ali, R.R., Reichel, M.B., De Alwis, M., Kanuga, N., Kinnon, C., Levinsky, R.J., Hunt, D.M., Bhattacharya, S.S., and Thrasher, A.J. (1998). Adeno-associated virus gene transfer to mouse retina. Hum. Gene Ther. *9*, 81–86.

25. Li, Q., Miller, R., Han, P.Y., Pang, J., Dinculescu, A., Chiodo, V., and Hauswirth, W.W. (2008). Intraocular route of AAV2 vector administration defines humoral immune response and therapeutic potential. Mol. Vis. *14*, 1760–1769.

26. Dalkara, D., Byrne, L.C., Klimczak, R.R., Visel, M., Yin, L., Merigan, W.H., Flannery, J.G., and Schaffer, D.V. (2013). In Vivo-Directed Evolution of a New Adeno-Associated Virus for Therapeutic Outer Retinal Gene Delivery from the Vitreous. Sci. Transl. Med. *5*, 189ra76.

27. Prinz, M., and Priller, J. (2014). Microglia and brain macrophages in the molecular age: from origin to neuropsychiatric disease. Nat. Rev. Neurosci. *15*, 300–312.

28. Langmann, T. (2007). Microglia activation in retinal degeneration. J. Leukoc. Biol. *81*, 1345–1351.

29. Xiong, W., Wu, D.M., Xue, Y., Wang, S.K., Chung, M.J., Ji, X., Rana, P., Zhao, S.R., Mai, S., and Cepko, C.L. (2019). AAV *cis*-regulatory sequences are correlated with ocular toxicity. Proc. Natl. Acad. Sci. USA *116*, 5785–5794.

30. Bartel, M.A., Weinstein, J.R., and Schaffer, D.V. (2012). Directed evolution of novel adeno-associated viruses for therapeutic gene delivery. Gene Ther. *19*, 694–700.

31. Ross, M., and Ofri, R. (2021). The future of retinal gene therapy: evolving from subretinal to intravitreal vector delivery. Neural Regen. Res. *16*, 1751–1759.

32. Nickells, R.W., Schmitt, H.M., Maes, M.E., and Schlamp, C.L. (2017). AAV2-Mediated Transduction of the Mouse Retina After Optic Nerve Injury. Invest. Ophthalmol. Vis. Sci. *58*, 6091–6104.

33. Li, Y., Schlamp, C.L., and Nickells, R.W. (1999). Experimental induction of retinal ganglion cell death in adult mice. Invest. Ophthalmol. Vis. Sci. *40*, 1004–1008.

34. Schlamp, C.L., Montgomery, A.D., Mac Nair, C.E., Schuart, C., Willmer, D.J., and Nickells, R.W. (2013). Evaluation of the percentage of ganglion cells in the ganglion cell layer of the rodent retina. Mol. Vis. *19*, 1387–1396.

35. Nadal-Nicolás, F.M., Jiménez-López, M., Salinas-Navarro, M., Sobrado-Calvo, P., Vidal-Sanz, M., and Agudo-Barriuso, M. (2017). Microglial dynamics after axotomy-induced retinal ganglion cell death. J. Neuroinflammation *14*, 218.

36. Chang, B., Hawes, N.L., Hurd, R.E., Davisson, M.T., Nusinowitz, S., and Heckenlively, J.R. (2002). Retinal degeneration mutants in the mouse. Vision Res. *42*, 517–525.

37. Gargini, C., Terzibasi, E., Mazzoni, F., and Strettoi, E. (2007). Retinal organization in the retinal degeneration 10 (rd10) mutant mouse: a morphological and ERG study. J. Comp. Neurol. *500*, 222–238.

38. Summerford, C., and Samulski, R.J. (1998). Membrane-associated heparan sulfate proteoglycan is a receptor for adeno-associated virus type 2 virions. J. Virol. *72*, 1438–1445.

39. Ng, R., Govindasamy, L., Gurda, B.L., McKenna, R., Kozyreva, O.G., Samulski, R.J., Parent, K.N., Baker, T.S., and Agbandje-McKenna, M. (2010). Structural characterization of the dual glycan binding adeno-associated virus serotype 6. J. Virol. *84*, 12945–12957.

40. Boye, S.L., Bennett, A., Scalabrino, M.L., McCullough, K.T., Van Vliet, K., Choudhury, S., Ruan, Q., Peterson, J., Agbandje-McKenna, M., and Boye, S.E. (2016). Impact of Heparan Sulfate Binding on Transduction of Retina by Recombinant Adeno-Associated Virus Vectors. J. Virol. *90*, 4215–4231.

41. Wu, Z., Asokan, A., Grieger, J.C., Govindasamy, L., Agbandje-McKenna, M., and Samulski, R.J. (2006). Single amino acid changes can influence titer, heparin binding, and tissue tropism in different adeno-associated virus serotypes. J. Virol. *80*, 11393–11397.

42. Xie, Q., Lerch, T.F., Meyer, N.L., and Chapman, M.S. (2011). Structure-function analysis of receptor-binding in adeno-associated virus serotype 6 (AAV-6). Virology *420*, 10–19.

43. Dalkara, D., Kolstad, K.D., Caporale, N., Visel, M., Klimczak, R.R., Schaffer, D.V., and Flannery, J.G. (2009). Inner limiting membrane barriers to AAV-mediated retinal transduction from the vitreous. Mol. Ther. *17*, 2096–2102.

44. Matcovitch-Natan, O., Winter, D.R., Giladi, A., Vargas Aguilar, S., Spinrad, A., Sarrazin, S., Ben-Yehuda, H., David, E., Zelada González, F., Perrin, P., et al. (2016). Microglia development follows a stepwise program to regulate brain homeostasis. Science *353*, aad8670.

45. Li, X.G., Okada, T., Kodera, M., Nara, Y., Takino, N., Muramatsu, C., Ikeguchi, K., Urano, F., Ichinose, H., Metzger, D., et al. (2006). Viral-mediated temporally controlled dopamine production in a rat model of Parkinson disease. Mol. Ther. *13*, 160–166.

46. Zhao, X.F., Alam, M.M., Liao, Y., Huang, T., Mathur, R., Zhu, X., and Huang, Y. (2019). Targeting microglia using Cx3cr1-Cre lines: Revisiting the specificity. eNeuro *6*, 1–11.

47. Bauer, J., Sminia, T., Wouterlood, F.G., and Dijkstra, C.D. (1994). Phagocytic activity of macrophages and microglial cells during the course of acute and chronic relapsing experimental autoimmune encephalomyelitis. J. Neurosci. Res. *38*, 365–375.

48. Sholl, D.A. (1953). Dendritic organization in the neurons of the visual and motor cortices of the cat. J. Anat. *87*, 387–406.

49. Fischer, K.B., Collins, H.K., and Callaway, E.M. (2019). Sources of off-target expression from recombinasedependent AAV vectors and mitigation with cross-over insensitive ATG-out vectors. Proc. Natl. Acad. Sci. USA *116*, 27001–27010.

50. Saunders, A., and Sabatini, B.L. (2015). Cre Activated and Inactivated Recombinant Adeno-Associated Viral Vectors for Neuronal Anatomical Tracing or Activity Manipulation. Curr. Protoc. Neurosci. *72*, 1.24.1–1.24.15.

51. Madisen, L., Zwingman, T.A., Sunkin, S.M., Oh, S.W., Zariwala, H.A., Gu, H., Ng, L.L., Palmiter, R.D., Hawrylycz, M.J., Jones, A.R., et al. (2010). A robust and high-throughput Cre reporting and characterization system for the whole mouse brain. Nat. Neurosci. *13*, 133–140.

52. Sarra, G.M., Stephens, C., Schlichtenbrede, F.C., Bainbridge, J.W.B., Thrasher, A.J., Luthert, P.J., and Ali, R.R. (2002). Kinetics of transgene expression in mouse retina following sub-retinal injection of recombinant adeno-associated virus. Vision Res. *42*, 541–549.

53. McCarty, D.M. (2008). Self-complementary AAV vectors; advances and applications. Mol. Ther. *16*, 1648–1656.

54. Woodard, K.T., Liang, K.J., Bennett, W.C., and Samulski, R.J. (2016). Heparan Sulfate Binding Promotes Accumulation of Intravitreally Delivered Adeno-associated Viral Vectors at the Retina for Enhanced Transduction but Weakly Influences Tropism. J. Virol. *90*, 9878–9888.

55. Halbert, C.L., Allen, J.M., and Miller, A.D. (2001). Adeno-associated virus type 6 (AAV6) vectors mediate efficient transduction of airway epithelial cells in mouse lungs compared to that of AAV2 vectors. J. Virol. *75*, 6615–6624.

56. Pellissier, L.P., Hoek, R.M., Vos, R.M., Aartsen, W.M., Klimczak, R.R., Hoyng, S.A., Flannery, J.G., and Wijnholds, J. (2014). Specific tools for targeting and expression in Müller glial cells. Mol. Ther. Methods Clin. Dev. *1*, 14009.

57. Cao, X., Yung, J., Mak, H., and Leung, C.K.S. (2019). Factors governing the transduction efficiency of adeno-associated virus in the retinal ganglion cells following intravitreal injection. Gene Ther. *26*, 109–120.

58. Uren, P.J., Lee, J.T., Doroudchi, M.M., Smith, A.D., and Horsager, A. (2014). A profile of transcriptomic changes in the rd10 mouse model of retinitis pigmentosa. Mol. Vis. *20*, 1612–1628.

59. Keren-Shaul, H., Spinrad, A., Weiner, A., Matcovitch-Natan, O., Dvir-Szternfeld, R., Ulland, T.K., David, E., Baruch, K., Lara-Astaiso, D., Toth, B., et al. (2017). A Unique Microglia Type Associated with Restricting Development of Alzheimer's Disease. Cell *169*, 1276–1290.e17.

60. Åkerblom, M., Sachdeva, R., Quintino, L., Wettergren, E.E., Chapman, K.Z., Manfre, G., Lindvall, O., Lundberg, C., and Jakobsson, J. (2013). Visualization and genetic modification of resident brain microglia using lentiviral vectors regulated by microRNA-9. Nat. Commun. *4*, 1770.

61. Sun, E., and Shi, Y. (2015). MicroRNAs: Small molecules with big roles in neurodevelopment and diseases. Exp. Neurol. *268*, 46–53.

62. Ye, Y., Xu, H., Su, X., and He, X. (2016). Role of MicroRNA in Governing Synaptic Plasticity. Neural Plast. *2016*, 4959523.

63. Wieghofer, P., Knobeloch, K.P., and Prinz, M. (2015). Genetic targeting of microglia. Glia *63*, 1–22.

64. Chistiakov, D.A., Killingsworth, M.C., Myasoedova, V.A., Orekhov, A.N., and Bobryshev, Y.V. (2017). CD68/macrosialin: not just a histochemical marker. Lab. Invest. *97*, 4–13.

65. Su, N., März, S., Plagemann, T., Cao, J., Schnittler, H.J., Eter, N., and Heiduschka, P. (2019). Occurrence of transmembrane protein 119 in the retina is not restricted to the microglia: An immunohistochemical study. Transl. Vis. Sci. Technol. *8*, 29.

66. Klawonn, A.M., Fritz, M., Castany, S., Pignatelli, M., Canal, C., Similä, F., Tejeda, H.A., Levinsson, J., Jaarola, M., Jakobsson, J., et al. (2021). Microglial activation elicits a negative affective state through prostaglandin-mediated modulation of striatal neurons. Immunity *54*, 225–234.e6.

67. Nie, X., Kitaoka, S., Tanaka, K., Segi-Nishida, E., Imoto, Y., Ogawa, A., Nakano, F., Tomohiro, A., Nakayama, K., Taniguchi, M., et al. (2018). The Innate Immune Receptors TLR2/4 Mediate Repeated Social Defeat Stress-Induced Social Avoidance through Prefrontal Microglial Activation. Neuron *99*, 464–479.e7.

68. Masuda, T., Sankowski, R., Staszewski, O., and Prinz, M. (2020). Microglia Heterogeneity in the Single-Cell Era. Cell Rep. *30*, 1271–1281.

69. Ronning, K.E., Karlen, S.J., Miller, E.B., and Burns, M.E. (2019). Molecular profiling of resident and infiltrating mononuclear phagocytes during rapid adult retinal degeneration using single-cell RNA sequencing. Sci. Rep. *9*, 4858.

70. Scholz, R., Caramoy, A., Bhuckory, M.B., Rashid, K., Chen, M., Xu, H., Grimm, C., and Langmann, T. (2015). Targeting translocator protein (18 kDa) (TSPO) dampens pro-inflammatory microglia reactivity in the retina and protects from degeneration. J. Neuroinflammation *12*, 201.

71. Scholz, R., Sobotka, M., Caramoy, A., Stempfl, T., Moehle, C., and Langmann, T. (2015). Minocycline counter-regulates pro-inflammatory microglia responses in the retina and protects from degeneration. J. Neuroinflammation *12*, 209.

72. Grieger, J.C., Choi, V.W., and Samulski, R.J. (2006). Production and characterization of adeno-associated viral vectors. Nat. Protoc. *1*, 1412–1428.

73. Pfaffl, M.W. (2004). Quantification strategies in real-time PCR. In A–Z of Quantitative PCR, S.A. Bustin, ed. (International University Line), pp. 87–112.

74. Bronstein, R., Torres, L., Nissen, J.C., and Tsirka, S.E. (2013). Culturing microglia from the neonatal and adult central nervous system. J. Vis. Exp. (78), 50647.

75. Wang, Z., Ma, H.-I., Li, J., Sun, L., Zhang, J., and Xiao, X. (2003). Rapid and highly efficient transduction by double-stranded adeno-associated virus vectors in vitro and in vivo. Gene Ther *10*, 2105–2111.

OMTM, Volume *23*

Supplemental information

Optimizing AAV2/6 microglial targeting identified enhanced efficiency in the photoreceptor degenerative environment

Margaret E. Maes, Gabriele M. Wögenstein, Gloria Colombo, Raquel Casado-Polanco, and Sandra Siegert

Figure S1

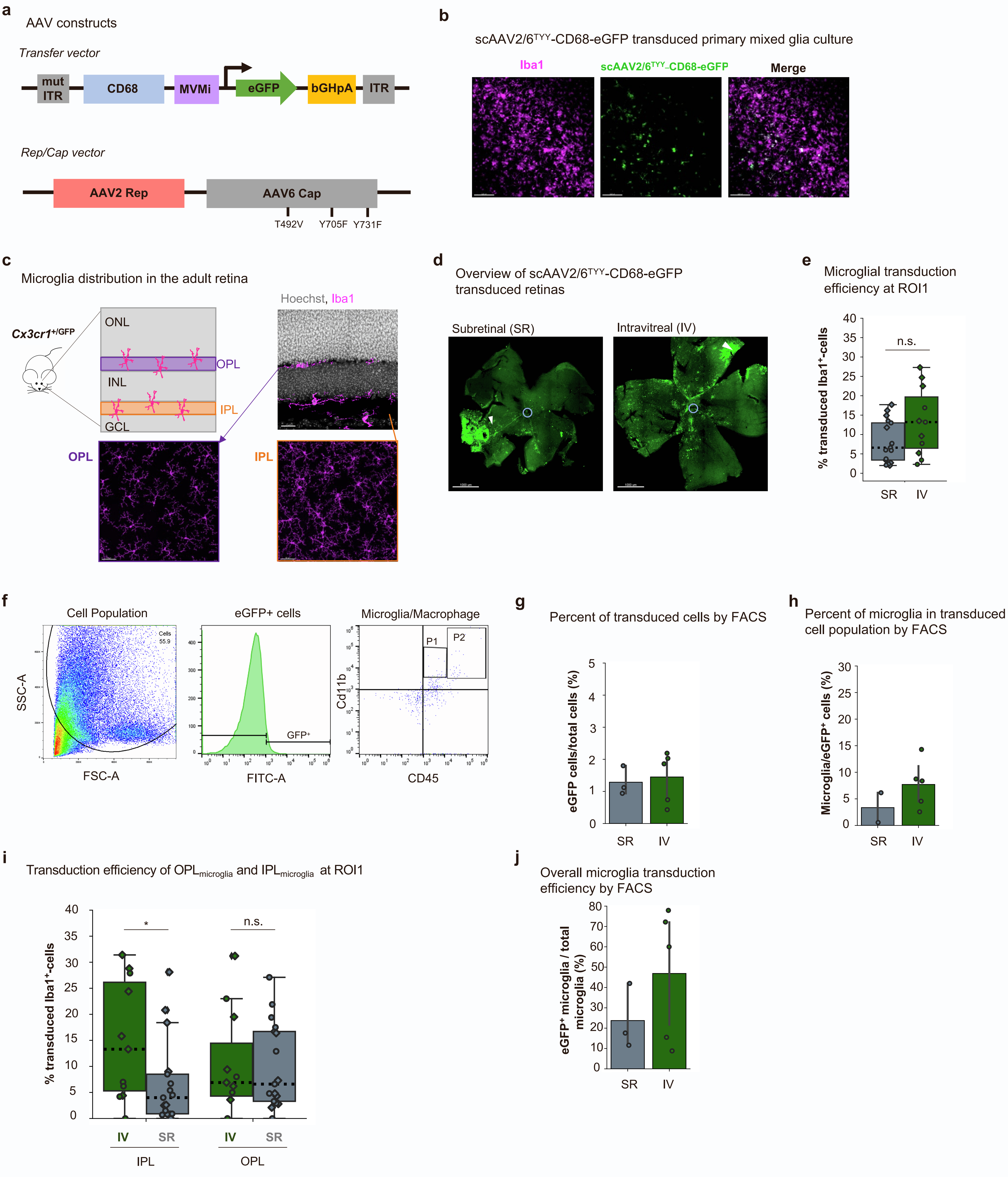

Figure S1. scAAV2/6^{TYY}-CD68-eGFP design and validation *in vitro* and *in vivo*

(**a**) Transfer vector illustration containing the *CD68* promoter, MVM intron and Rep/Cap vector with indicated TYY mutations in capsid sequence. (**b**) Mixed primary glia cultures transduced with 1*10^{8} viral genomes/well of scAAV2/6^{TYY}-CD68-eGFP (green) counterstained with Iba1 (magenta). Scale bar: 200µm. (**c**) Pictorial representation of the retinal layers, highlighting the outer plexiform layer (OPL, purple) and inner plexiform layer (IPL, orange), with corresponding confocal images of retinal cross section or whole mount images of Iba1-stained microglia (magenta). Scale bar: 50µm. (**d**) Overview images of retinas from subretinal (SR) or intravitreal (IV) injection indicating viral-mediated eGFP transgene expression. Blue circle indicates optic nerve head. White triangles: injection site. (**e**) Microglial transduction efficiency from quantification from ROI1 in both plexiform layers using 20X images. (Wilcoxon rank-sum test: P = 0.011). Scale bar: 1000µm. (**f**) Gating strategy for FACS sorted eGFP^{+} cells from a transduced retina. Microglia (P1) are CD11b^{hi}/CD45^{lo}, while macrophages (P2) are CD11b^{hi}/CD45^{hi}. (**g**) Percentage of eGFP^{+} cells in total cell population or (**h**) percent of transduced eGFP^{+} cells that are microglia in intravitreal and subretinal injected retinas. (IV: n= 5 retinas, 3 mice; SR: n = 3 retinas, 3 mice) (**i**) Inter-layer comparison of microglia transduction efficiency at ROI1. (Wilcoxon rank-sum test: IPL SR-IV, P = 0.048; OPL SR-IV, P = 0.79). (**j**) Overall microglia transduction efficiency. Percent of eGFP^{+} CD11b^{hi}/CD45^{lo} microglia / total CD11b^{hi}/CD45^{lo} microglia. *P < 0.05, ^{ns}P > 0.05. ITR, inverted terminal repeat; *CD68*, Cluster of differentiation 68; MVMi, minute virus of mice intron; eGFP, enhanced green fluorescent protein; bGHpA, bovine growth hormone polyadenylation signal; Rep, Replication; Cap, Capsid; scAAV, self-complementary adeno-associated virus; Iba1, ionized calcium binding adaptor molecule 1; ONL, outer nuclear layer; OPL, outer plexiform layer INL, inner nuclear layer; IPL, inner plexiform layer; GCL, ganglion cell layer; Cx3cr1, CX3C chemokine receptor 1; SR, subretinal; IV, intravitreal; P, postnatal day; ROI, region of interest; n.s., not significant.

Figure S2

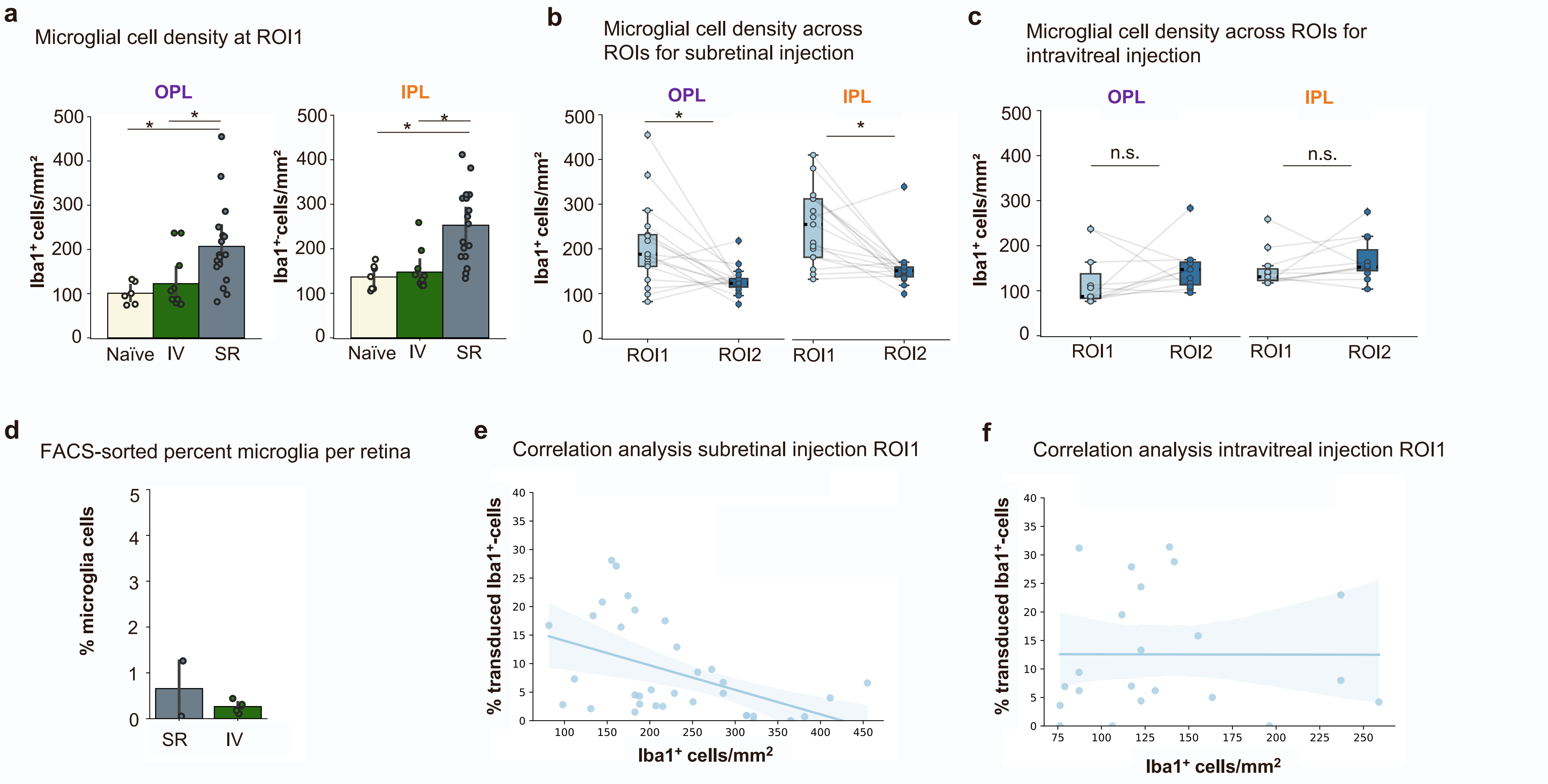

Figure S2. Microglial cell density after viral injection

(**a**) Quantification of $OPL_{microglia}$ and $IPL_{microglia}$ cell densities per mm^2 in retinas from naïve (un-injected), intravitreal or subretinal injections at ROI1. (Kruskal-wallis test: OPL, *P* = 0.0002; IPL, *P* = 0.003; post-hoc comparisons Wilcoxon rank-sum test: OPL SR-Naïve, *P* = 0.002; OPL SR-IV, *P* = 0.0004; IPL SR-Naïve, *P* = 0.004; IPL SR-IV, *P* = 0.010). (**b, c**) Comparison of microglial density between ROIs for both OPL and IPL niche after subretinal (Wilcoxon signed-rank test: OPL, *P* = 0.004; *IPL, P* = 0.004) or intravitreal injections (**c,** Wilcoxon signed-rank test: OPL*, P* = 0.248; *IPL, P* = 0.153). (**b**) Percent of microglia ($CD11b^{hi}/CD45^{lo}$) in total cell population of FACS-sorted IV or SR injected retinas. (**e, f**) Correlation using linear regression model between microglial cell density and transduction efficiency for subretinal (*Pearson's r* = -0.476, *P* = 0.004) or (**e**) intravitreal injection (*Pearson's r* = 0.002, *P* = 0.992). $^{*}P < 0.05$, $^{ns}P >$ 0.05. OPL, outer plexiform layer; IPL, inner plexiform layer; ROI, region of interest. Iba1, ionized calcium binding adaptor molecule 1; SR, subretinal; IV, intravitreal; n.s., not significant.

Figure S3

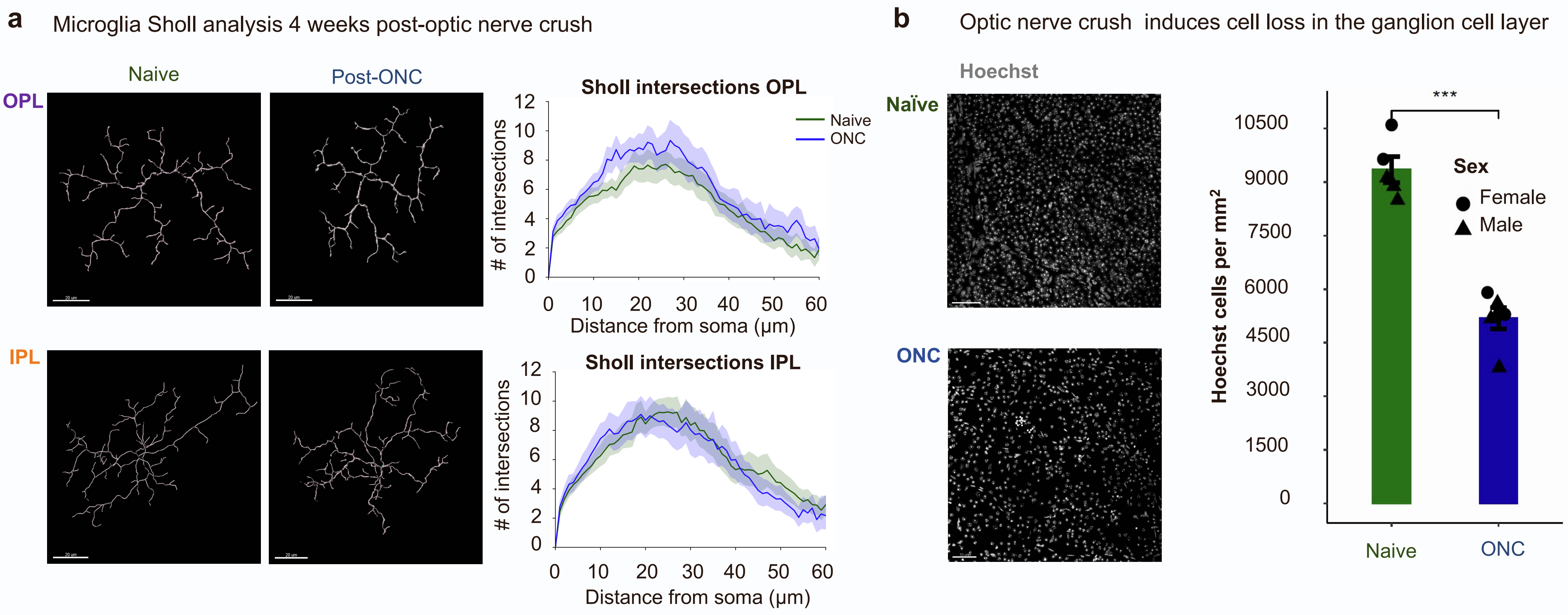

Figure S3. Microglial morphology and ganglion cell loss four weeks after optic nerve crush
(**a**) Example microglial tracings for naïve or four weeks post-optic nerve crush in OPL or IPL. Sholl intersections quantification from 40x 2x2 tiled images from >18 cells from at least 2 animals for each layer and condition. Scale bar: 20µm. (**b**) Hoechst-stained retinal whole mount images of ganglion cell layer (GCL). Total number of nuclei in the GCL is reduced in ONC. Scale bar: 50µm (Welch's Two-sample t-test: $P = 0.00001$). ***P< 0.005. OPL, outer plexiform layer; IPL, inner plexiform layer; ONC, optic nerve crush;

Figure S4

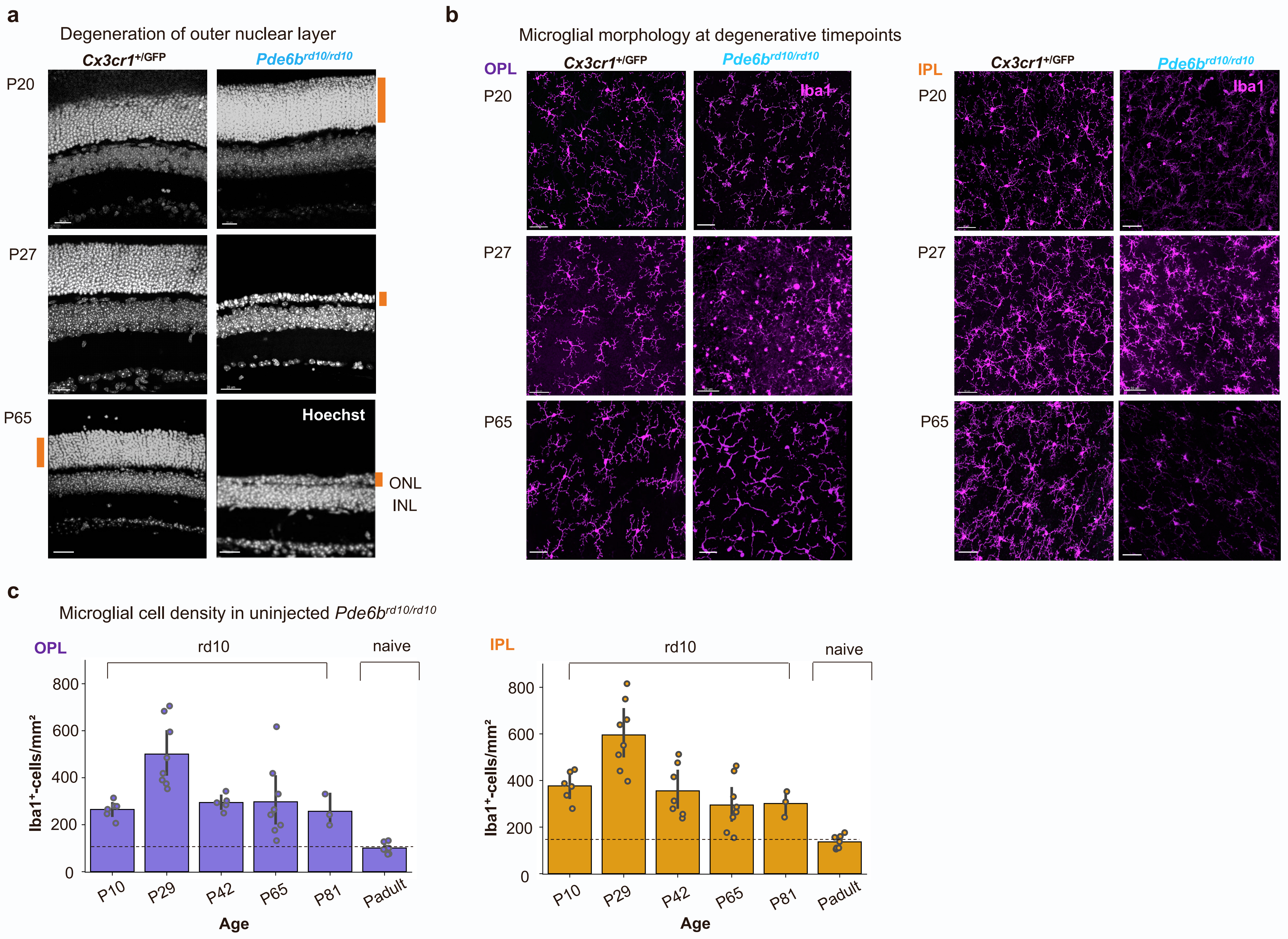

Figure S4. ONL thinning and microglial phenotype in *Pde6b*$^{rd10/rd10}$ degeneration model
(**a**) Hoechst-labeled retinal cross sections from control *Cx3cr1*$^{GFP/+}$ or *Pde6b*$^{rd10/rd10}$ mice at P20, P27 and P65. Orange bar highlights ONL thickness. Scale bar: 20µm. (**b**) Retinal wholemounts immunostained with Iba1 (magenta) depicting $OPL_{microglia}$ or $IPL_{microglia}$ in control *Cx3cr1*$^{GFP/+}$ or *Pde6b*$^{rd10/rd10}$ mice. Scale bar: 50µm. (**c**) Microglial cell density in OPL or IPL of uninjected *Pde6b*$^{rd10/rd10}$ compared to naïve (dashed line) adult mice. (**d**) Cell density at ROI1 of OPL or IPL microglia in P65 *Pde6b*$^{rd10/rd10}$ retinas injected with scAAV2/6^{TYY}-CD68-eGFP. Cx3cr1, CX3C chemokine receptor 1; ONL, outer nuclear layer; INL, inner nuclear layer. P, postnatal day; Iba1, ionized calcium binding adaptor molecule 1; OPL, outer plexiform layer; IPL, inner plexiform layer; eGFP, enhanced green fluorescent protein; *CD68*, Cluster of differentiation 68.

Figure S5

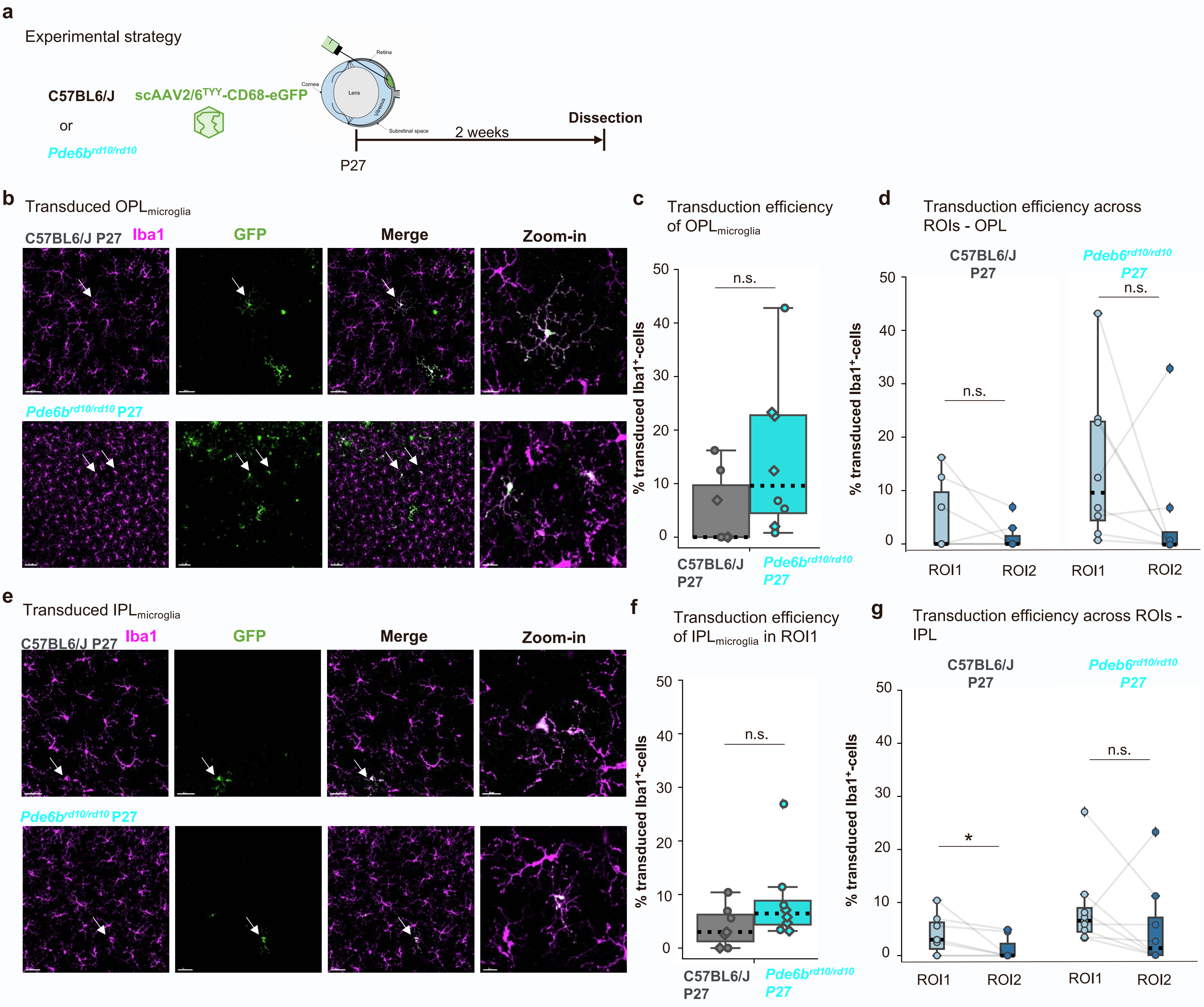

Figure S5. ONL loss in P27 *Pde6b$^{rd10/rd10}$* benefits OPL microglia

(**a**) Experimental timeline. *Pde6b$^{rd10/rd10}$* or C57BL6/J mice received subretinal injection of scAAV2/6^{TYY}-CD68-eGFP (1.37*10^{11}gc/mL) at postnatal day 27 (P27) and retinas were collected 2 weeks after injection. (**b, e**) Retinal wholemounts of OPL$_{microglia}$ (**b**) and IPL$_{microglia}$ (**e**) from indicated strain after subretinal injection stained with Iba1 (magenta) and eGFP (green). White arrows indicate zoom-in region. Scale bar: 50µm, zoom-in: 15µm. (**c, f**) Percent transduction efficiency in OPL$_{microglia}$ (**c,** Wilcoxon ranked-sum test, *P*=0.105) and IPL$_{microglia}$ (**f,** Wilcoxon ranked-sum test, *P*=0.132) Stats. (**d, g**) Transduction across ROIs in C57BL6/J and P27 *Pde6b$^{rd10/rd10}$* retinas in the OPL (**d,** Wilcoxon signed-rank test: C57BL6/J, *P* = 0.144; *Pde6b$^{rd10/rd10}$*, *P* = 0.123) and IPL (**g,** Wilcoxon signed-rank test: C57BL6/J, *P* = 0.043; *Pde6b$^{rd10/rd10}$*, *P* = 0.161). *Pde6b$^{rd10/rd10}$*: n= 8 retinas, 7 mice. C57BL6/J: n= 7 retinas, 4 mice. *$P < 0.05$, $^{ns}P > 0.05$. scAAV, self-complementary adeno-associated virus; P, postnatal day; Iba1, ionized calcium binding adaptor molecule 1; GFP, green fluorescent protein; eGFP, enhanced green fluorescent protein; *CD68*, Cluster of differentiation 68; OPL, outer plexiform layer; IPL, inner plexiform layer; ROI, region of interest; n.s., not significant.

Figure S6

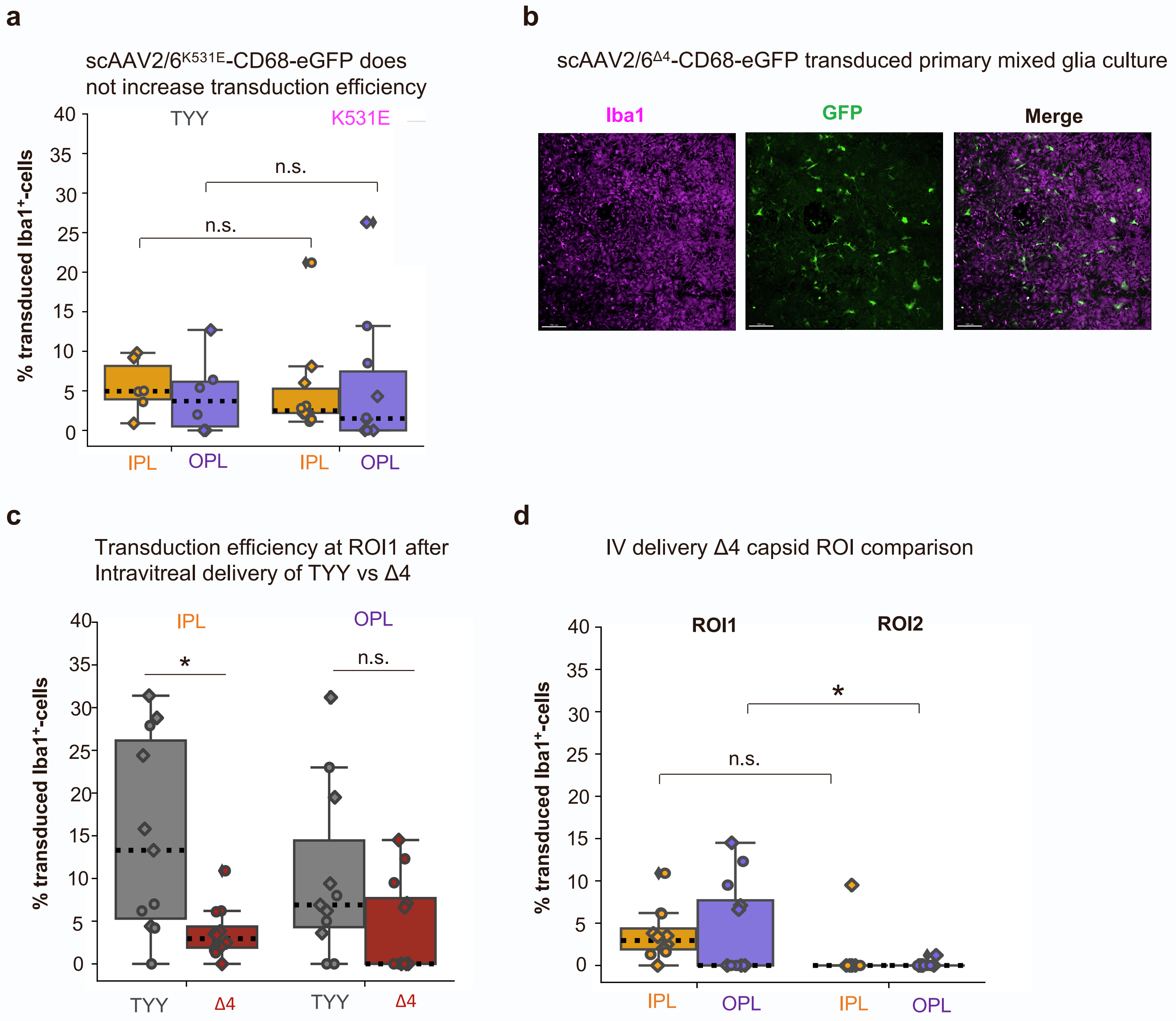

Figure S6. Single heparin-binding mutant and intravitreal delivery of AAV2/6 $^{\Delta4}$
(**a**) Single capsid mutant scAAV2/6^{K531E}-CD68-eGFP microglial transduction efficiency compared to scAAV2/6^{TYY}-CD68-eGFP (Wilcoxon ranked-sum test, OPL, P = 0.828; IPL, P = 0.329). (**b**) Mixed primary glia cultures transduced with 1*10^{8} viral genomes/well of scAAV2/6$^{\Delta4}$-CD68-eGFP counterstained with Iba1 (magenta). Scale bar: 200 µm. (**c**) Comparison of intravitreally delivered scAAV2/6^{TYY}-CD68-eGFP and scAAV2/6$^{\Delta4}$-CD68-eGFP transduction efficiency of OPL$_{microglia}$ and IPL$_{microglia}$ (Wilcoxon ranked-sum test: OPL, P = 0.139; IPL, P=0.005). (**d**) Comparison across ROIs for both OPL and IPL niche after intravitreal delivery of scAAV2/6$^{\Delta4}$-CD68-eGFP. TYY: 11 retinas, 6 mice. Δ4: 12 retinas, 6 mice. $^{*}P < 0.05$, $^{ns}P > 0.05$. scAAV, self-complementary adeno-associated virus; *CD68*, Cluster of differentiation 68; Iba1, ionized calcium binding adaptor molecule 1; IPL, inner plexiform layer; OPL, outer plexiform layer; GFP, green fluorescent protein; ROI, region of interest; TYY, scAAV2/6^{TYY}; Δ4, scAAV2/6$^{\Delta4}$; IV, intravitreal; n.s., not significant.

Figure S7

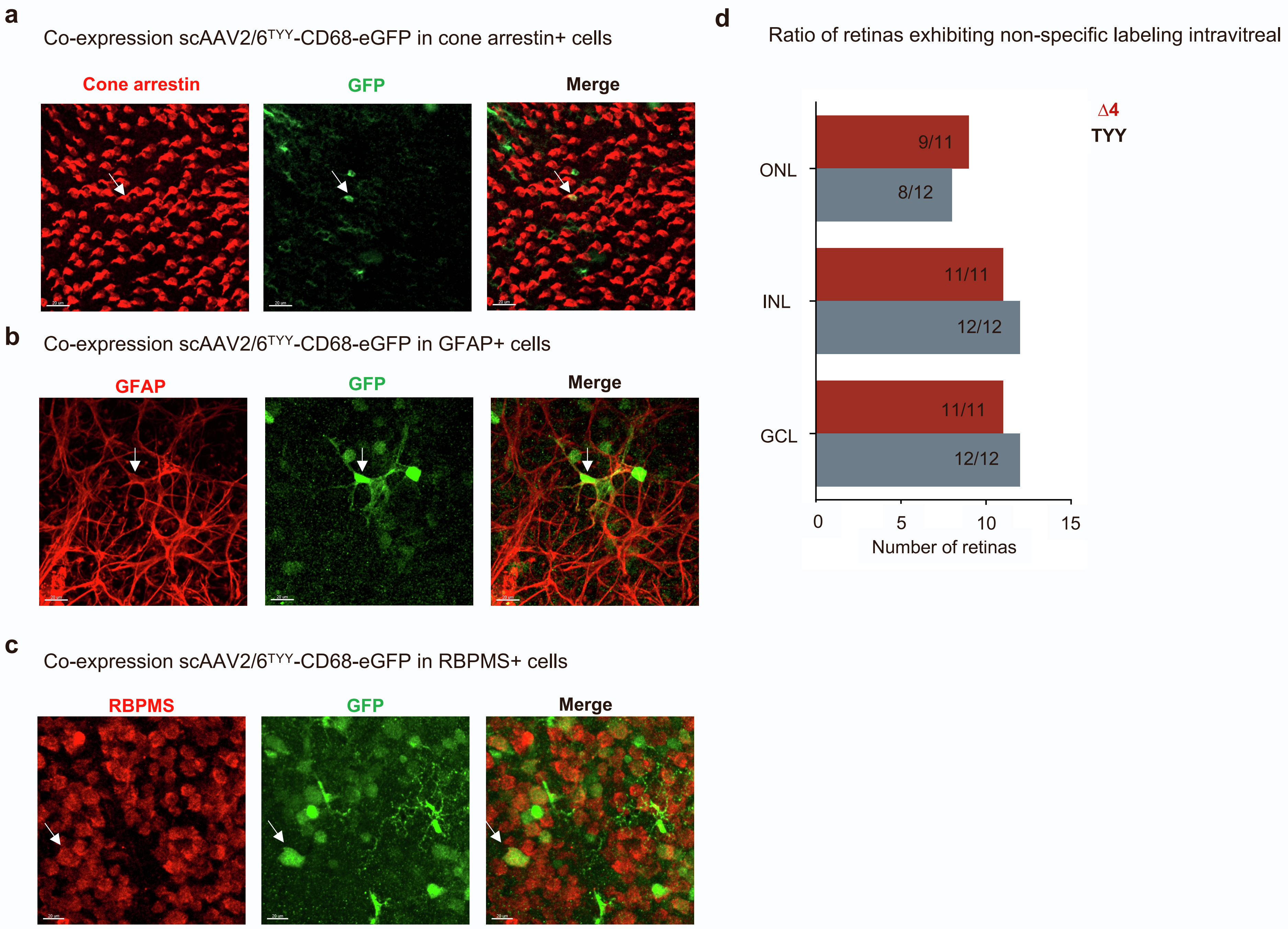

Figure S7. Non-specific labeling using scAAV2/6^{TYY}-CD68-eGFP

Retinal wholemounts of (**a**) cone photoreceptors (cone arrestin^{+}, red), (**b**) astrocytes (GFAP^{+}, red), (**c**) retinal ganglion cells (RPBMS^{+}) co-expressing eGFP (green) following subretinal injection of scAAV2/6^{TYY}-CD68-eGFP ($1*10^{12}$ gc/mL). White arrow indicates co-localized cell. Scale bar: 50µm. (**d**) Ratio of analyzed retinas exhibiting non-specific eGFP expression after subretinal delivery of scAAV2/6^{TYY}-CD68-eGFP across the nuclear layer, outer nuclear layer (ONL), inner nuclear layer (INL) and ganglion cell layer (GCL). scAAV, self-complementary adeno-associated virus; CD68, Cluster of differentiation 68; eGFP, enhanced green fluorescent protein; GFP, green fluorescent protein; GFAP, Glial Fibrillary Acidic Protein; RBPMS, RNA binding protein with multiple splicing; ONL, outer nuclear layer; INL, inner nuclear layer; GCL, ganglion cell layer; TYY, scAAV2/6^{TYY}; Δ4, scAAV2/6^{Δ4}.

Figure S8

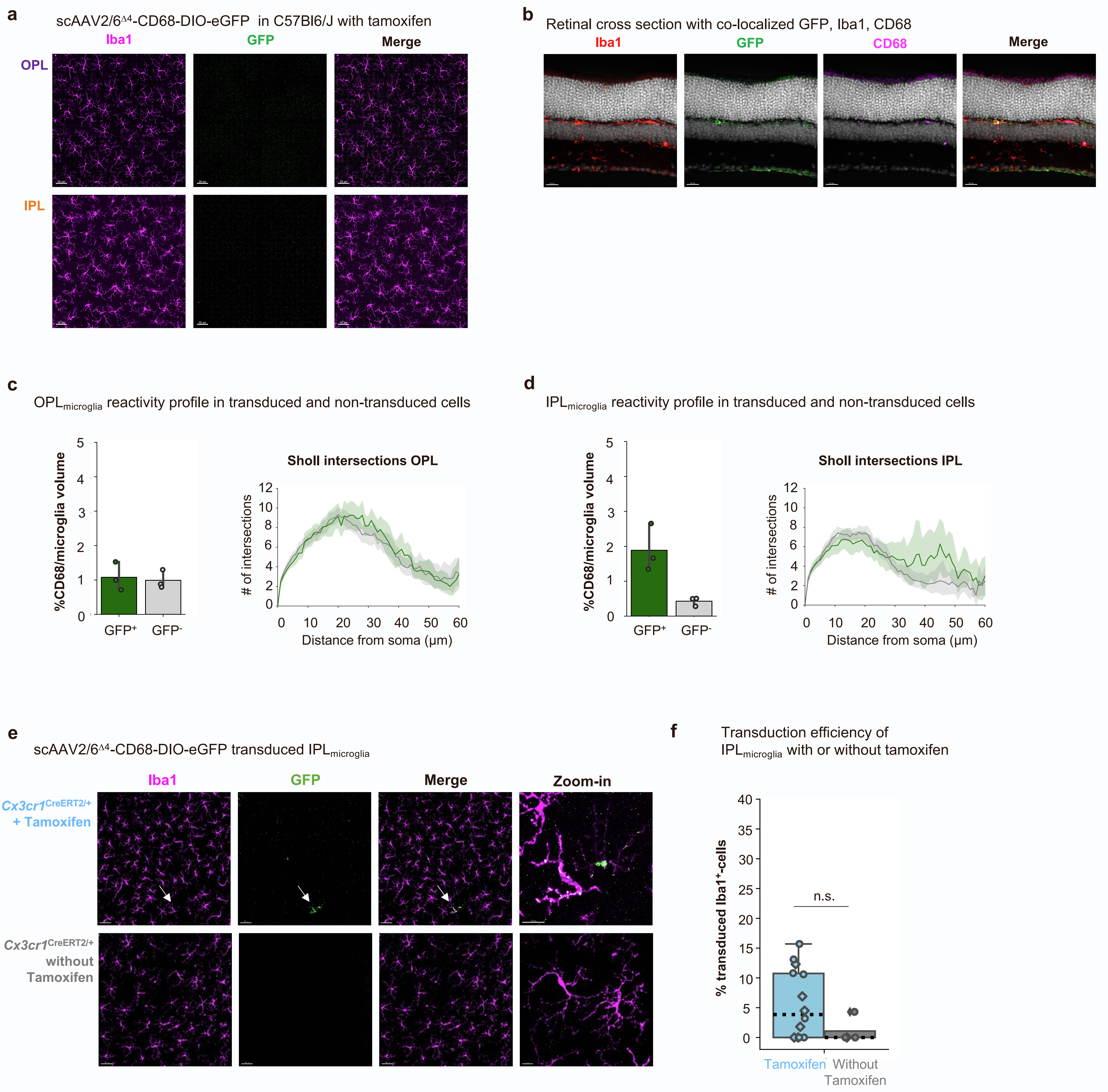

Figure S8. IPL$_{microglia}$ transduction after scAAV$^{\Delta4}$-CD68-DIO-eGFP delivery
(**a**) Retinal wholemounts of OPL and IPL microglia of C57BL6/J mice after subretinal delivery of scAAV$^{\Delta4}$-CD68-DIO-eGFP and tamoxifen treatment immunostained with Iba1 (magenta) and eGFP (green). Scale bar: 50µm. (**b**) Retinal cross section of scAAV2/6$^{\Delta4}$-CD68-DIO-eGFP injected in a *Cx3cr1*$^{CreERT2/+}$ mouse demonstrating colocalization of Iba1 (red), eGFP (green), CD68 (magenta). Nuclei stained with Hoechst (white). Scale bar: 20µm. (**c**) Quantification of % CD68 per microglia volume and Sholl analysis of cell morphology in transduced or untransduced OPL$_{microglia}$ and (**d**) IPL$_{microglia}$. (OPL: n = 47 cells non-transduced, n=15 cells transduced; 3 retinas; IPL: n = 44 cells non-transduced, n=15 cells transduced; 4 retinas) (**e**) Retinal wholemounts of in *Cx3cr1*$^{CreERT2/+}$ mice after subretinal delivery of scAAV$^{\Delta4}$-CD68-DIO-eGFP with or without tamoxifen induction immunostained with Iba1 (magenta) and eGFP (green). White arrows indicate zoom-in. Scale bar: 50µm, zoom-in: 15µm. (**e**) Comparison of transduction efficiency in the IPL with and without tamoxifen (Wilcoxon ranked-sum test: $P = 0.151$). C57BL6/J: 12 retinas, 7 mice. *Cx3cr1*$^{CreERT2/+}$ with Tam: 14 retinas, 9 mice. *Cx3cr1*$^{CreERT2/+}$ without Tam: 4 retinas, 3 mice. $^{*}P < 0.05$, $^{ns}P > 0.05$. scAAV, self-complementary adeno-associated virus; *CD68*, Cluster of differentiation 68; GFP, green fluorescent protein; eGFP, enhanced green fluorescent protein; Iba1, ionized calcium binding adaptor molecule 1; IPL, inner plexiform layer; OPL, outer plexiform layer; Cx3cr1, CX3C chemokine receptor 1; DIO, double-floxed inverse orientation; CreERT2, tamoxifen-inducible Cre recombinase; Tam, Tamoxifen; n.s., not significant.
